# Reprogramming Synthetic Cells for Targeted Cancer Therapy

**DOI:** 10.1021/acssynbio.1c00631

**Published:** 2022-03-08

**Authors:** Boon Lim, Yutong Yin, Hua Ye, Zhanfeng Cui, Antonis Papachristodoulou, Wei E. Huang

**Affiliations:** † Department of Engineering Science, University of Oxford, Parks Road, OX1 3PJ Oxford, U.K.; ‡ Institute of Biomedical Engineering, Department of Engineering Science, University of Oxford, OX3 7DQ Oxford, U.K.

**Keywords:** synthetic biology, SimCells, bacterial therapy, chromosome-free, I-CeuI endonuclease, minicells, drug delivery, catechol, cancer

## Abstract

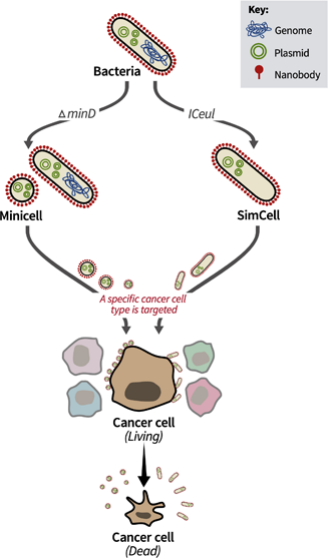

Advances in synthetic biology enable the reprogramming of bacteria as smart agents to specifically target tumors and locally release anticancer drugs in a highly controlled manner. However, the bench-to-bedside translation of engineered bacteria is often impeded by genetic instability and the potential risk of uncontrollable replication of engineered bacteria inside the patient. SimCells (simple cells) are chromosome-free bacteria controlled by designed gene circuits, which can bypass the interference of the native gene network in bacteria and eliminate the risk of bacterial uncontrolled growth. Here, we describe the reprogramming of SimCells and mini-SimCells to serve as “safe and live drugs” for targeted cancer therapy. We engineer SimCells to display nanobodies on the surface for the binding of carcinoembryonic antigen (CEA), which is an important biomarker found commonly in colorectal cancer cells. We show that SimCells and mini-SimCells with surface display of anti-CEA nanobody can specifically bind CEA-expressing Caco2 cancer cells *in vitro* while leaving the non-CEA-expressing SW80 cancer cells untouched. These cancer-targeting SimCells and mini-SimCells induced cancer cell death *in vitro* by compromising the plasma membrane of cancer cells. The cancer-killing effect can be further enhanced by an aspirin/salicylate inducible gene circuit that converts salicylate into catechol, a potent anticancer. This work highlights the potential of SimCells and mini-SimCells for targeted cancer therapy and lays the foundation for the application of synthetic biology to medicine.

## Introduction

Advances in synthetic biology have promoted the application of engineered bacteria to cancer therapy (reviewed in^1−4^). Scientists can now engineer bacteria that have attenuated virulence,^5^ high tumor specificity,^6^ and strict control of drug expression coupled with precise delivery (reviewed in^7^) to unleash the full potential of bacterial therapy in cancer treatment. Recent examples also include the adaptation of programmable bacteria to exert anti-tumor immune responses via a stimulator of interferon genes (STING) agonist,^8^ CD47 nanobody^9^ and tumor neoepitope.^10,11^

So far, most of the bacteria-based strategies rely on the intrinsic ability of bacteria to colonize and proliferate at the tumor microenvironment, especially within the hypoxic and necrotic tumor core.^8,12,13^ However, previous clinical trials showed that the prominent tumor colonization effect observed in a mouse model does not always translate to another host, such as human and canine.^14,15^ To enhance tumor targeting, specific motifs such as affibody and ligand are engineered onto the bacterial outer membrane to achieve cell-specific delivery.^16,17^ Yet, not all cancer cell-specific markers have a naturally available ligand that can be readily folded and expressed onto the bacterial surface. In addition, random mutations might lead to the loss-of-function.^18^ The safety and growth control of living bacteria are the concerns of bacterial cancer therapy.

We aim to overcome these challenges by developing SimCell (simple cell) cancer therapy. Chromosome-free mini-SimCells^19^ and SimCells^20^ can be reprogrammed to serve as “safe, smart, and live drug” for targeted cancer therapy. Mini-SimCells are minicells containing designed gene circuits.^19^ Because of the small size (100–400 nm) of mini-SimCells, they can infiltrate deep to the tumor. Normal-size SimCells (in short SimCells) are chromosome-free bacterial cells containing designed gene circuits.^20^ SimCells can be generated from many different bacterial chassis, such as *Escherichia coli*, *Pseudomonas putida*, and *Ralstonia eutropha.*(20) The chassis we used in this study is *E. coli* BL21(DE3).^21^ SimCells are nonreplicating and highly controllable, taking advantage of the live and nonlive system, built from pre-existing bacterial cells, and hijacked by designed gene circuits to perform novel and safe tasks in cancer treatment.

Some tumor surface antigens can be used as a biomarker of cancers.^22,23^ We adopted a modular surface display system reported previously to express anti-biomarker nanobody^24^ on the outer membrane of engineered mini-SimCells and SimCells. The specificity of this system can be modulated by raising the nanobody against a marker of interest through an immune library.^25^ Various cytotoxic proteins (e.g., pore-forming hemolytic protein) and chemotherapeutic agents (e.g., 5-fluorouracil) can be used to attack tumors.^26^ An inducible gene circuit provides a controllable production and release of anticancer agents in tumor microenvironment.^7^ Due to the direct binding between SimCells and cancer cells,^20^ it would locally create a high dose of anticancer agents without harming other normal tissue.

In this study, we used carcinoembryonic antigen (CEA), one of the thoroughly studied tumor biomarkers in cancer diagnostics and monitoring, as our marker of interest.^27^ We engineered mini-SimCells and SimCells to display anti-CEA nanobodies on the surface that can specifically bind CEA-expressing Caco2, a colorectal cancer (CRC) cell line.^23,28^ Aspirin and salicylate are safe small molecules, able to induce a gene circuit^29^ that transform aspirin/salicylate into a potent anticancer compound—catechol.^20^ We evaluated both mini-SimCells and SimCells as stand-alone cancer therapeutics *in vitro* with and without the additional synthetic circuit for anticancer compound (catechol) synthesis. The chromosome-free and nondividing SimCells are safe and able to achieve controlled delivery of therapeutic payloads into the tumor microenvironment. Importantly, both chassis would have therapeutic potential for new bacteria-derived cancer treatment.

## Results

### A Biological Agglutination Test Confirms the Binding of Nanobody-Expressing *E. coli* to CEA

Surface displays of recombinant protein using bacterial outer membrane protein,^30^ lipoprotein,^31^ autotransporter protein,^32^ and ice nucleation protein^33^ have successfully led to the development of multiple biotechnological applications. Nevertheless, the binding of *E. coli* to a target surface will require a surface display system that has high expression efficiency and precise protein folding without compromising cell activity. In this study, we adopted a previously reported system using the β-intimin domain as the anchor^24,34−36^ to express nanobody on the surface of *E. coli* BL21(DE3), which has shown specific binding to CEA. We found that *E. coli* BL21(DE3) is able to perform a good nanobody surface display for the antigen-binding. It might be due to the fact that this protease-deficient strain is optimized for heterologous protein expression, and it has a minimal interference of flagella and fimbriae to small nanobodies (2–4 nm) on its surface.

**Figure 1:**
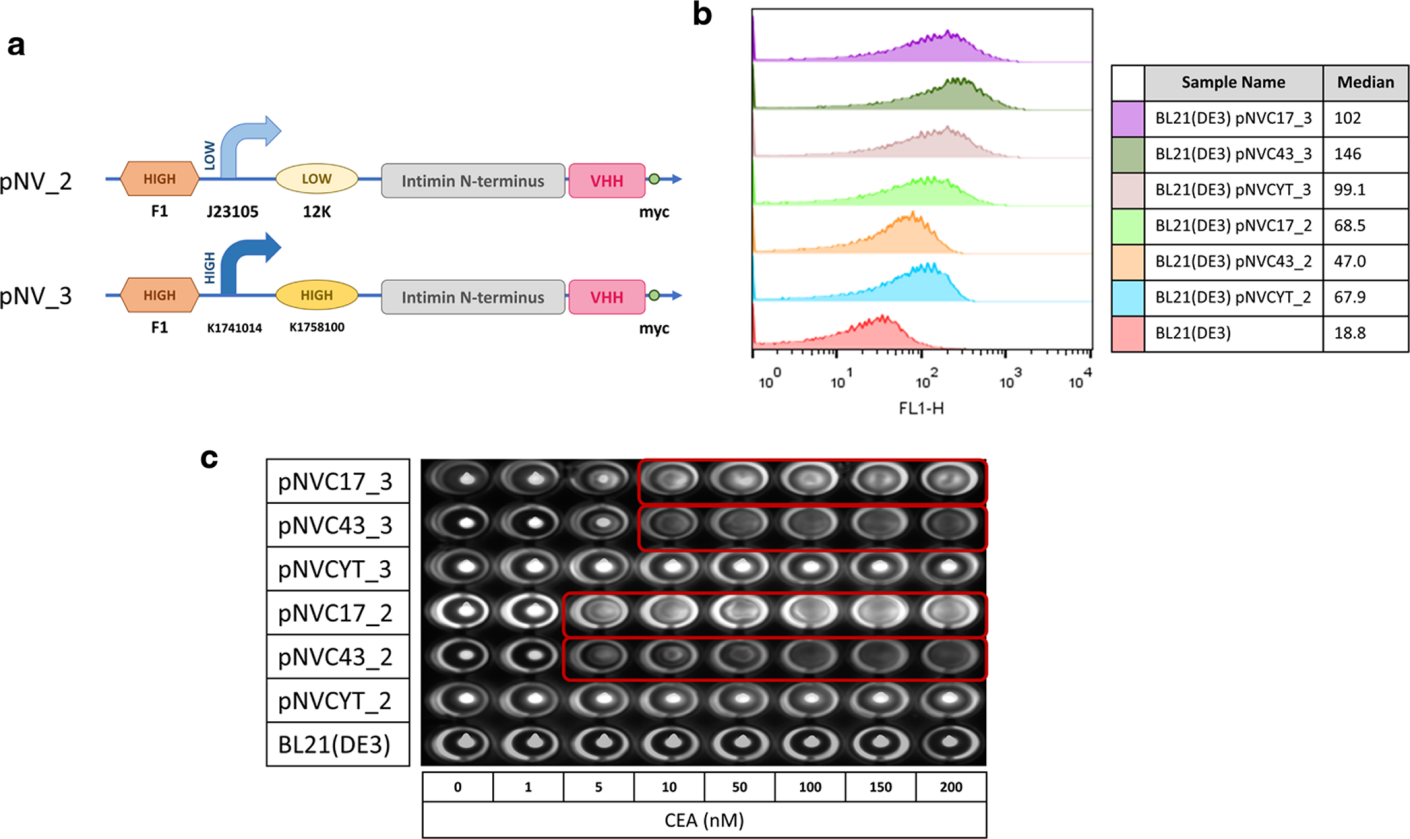
Engineered *E. coli* BL21(DE3) expressing nanobody through the surface display pNV system. (a) Schematic representation of pNV_2 with low expression profile and pNV_3 with high expression profile. F1 is a high copy number origin of replication; J23105 has low promoter activity, whereas K1741014 has high promoter activity; 12 k is a low strength Ribosome Binding Strength (RBS), whereas K1758100 is a high strength RBS; Intimin is the outer membrane anchor; VHH represents the nanobody used in this study, which includes anti-CEA, C17, and C43 or anti-spike protein, CYT; Myc is the tag used for flow cytometry analysis. (b) Flow cytometry analysis of engineered *E. coli* BL21(DE3) carrying different pNV plasmids. Sample BL21(DE3) represents wild-type bacteria without any plasmid. Histograms indicate the fluorescence intensity of bacteria probed with primary anti-Myc antibody and secondary Alexa Fluor 488 antibody. The median fluorescence intensity of each sample is also presented. (c) Binding of engineered *E. coli* BL21(DE3) to CEA in a biological agglutination test.For the test, 100 μL (OD_600_ = 0.5) of engineered *E. coli* BL21(DE3) carrying different pNV plasmids was added with a range of concentrations of CEA in a clear, round-bottom 96-well plate. Sample BL21(DE3) represents engineered *E. coli* carrying an empty pNV plasmid without the VHH region. Binding between surface-displayed nanobody and target antigen CEA results in agglutination (red box, clear suspension), while no binding results in a cell pellet. The image was taken using VersaDoc imaging system under FITC channel.

We first designed two surface display systems: (i) pNV_2 with a promoter of low expression strength J23105 and (ii) pNV_3 with a promoter of high expression strength K1758100 (http://parts.igem.org/Catalog) (Figure 1a) to accommodate the anti-CEA nanobody candidates, C17^37^ and C43.^38^ For a surface display control, we used an anti-spike protein nanobody, CYT,^39^ which is designed to target the receptor-binding domain of the SARS-CoV-2 virus. Surface display of the nanobodies on *E. coli* BL21(DE3) was confirmed by flow cytometry using a primary anti-Myc antibody and a secondary Alexa Fluor 488 conjugated antibody (Figure 1b). The nanobody expression of C17, C43, and CYT on the surface showed strong fluorescent signals compared to the wild-type control of *E. coli* BL21(DE3).

To investigate the CEA binding ability of the nanobodies, we first employed a biological agglutination test that resembles the clinically used latex agglutination test.^35,36^ Briefly, in the presence of target analyte/antigen, such as CEA in this case, surface-displayed nanobodies will cross-link with the target molecules, resulting in agglutination through a bacteria–target–bacteria sandwich interaction. When surface nanobodies bind with the target analyte/antigen, bacteria will create a cloudy or clear suspension; and without binding, bacteria will form cell pellets, which can be visually examined in a round-bottom 96-well plate.^29^ We set up the biological agglutination test following a standard diagnostic hemagglutination assay: a series of concentrations of human CEA protein ranging from 1 to 200 nM were added to the same concentration of bacterial cells carrying the pNV plasmids in a round-bottom 96-well plate. For ease of visualization, super folder green fluorescence protein (sfGFP) was cloned downstream to the nanobody in all pNV plasmids (Supporting Information Figure S1). After overnight incubation, cell agglutination was observed in cultures expressing C17 and C43 nanobodies within the range of 5–200 nM of CEA in pNV_2 and 10–200 nM of CEA in pNV_3 system, while none of the CYT controls showed any agglutination (Figure 1c). This result demonstrates the binding capacity of engineered *E. coli* surface-displaying C17 and C43 nanobodies to human CEA protein. Consistent with the previously reported model, modifying the surface expression level of nanobody will shift the equivalence zone of the assay, which results in different diagnostic sensitivity.^35,36^ In the following experiments, the pNV_3 system was chosen (named pNV_sfGFP from here onward, Supporting Information Figure S1), because it has a strong promoter for nanobody expression, increasing the binding possibility between surface-displaying nanobody on *E. coli* and CEA antigen on human cell lines.

### Engineered *E. coli* Specifically Binds to CEA-Expressing Colorectal Cancer Cells In Vitro

We tested the *in vitro* binding efficacy of engineered *E. coli* to two colorectal cancer cell lines with different CEA-expression strengths: (i) Caco2 with high CEA expression level and (ii) SW480 with low CEA expression level.^28^

**Figure 2:**
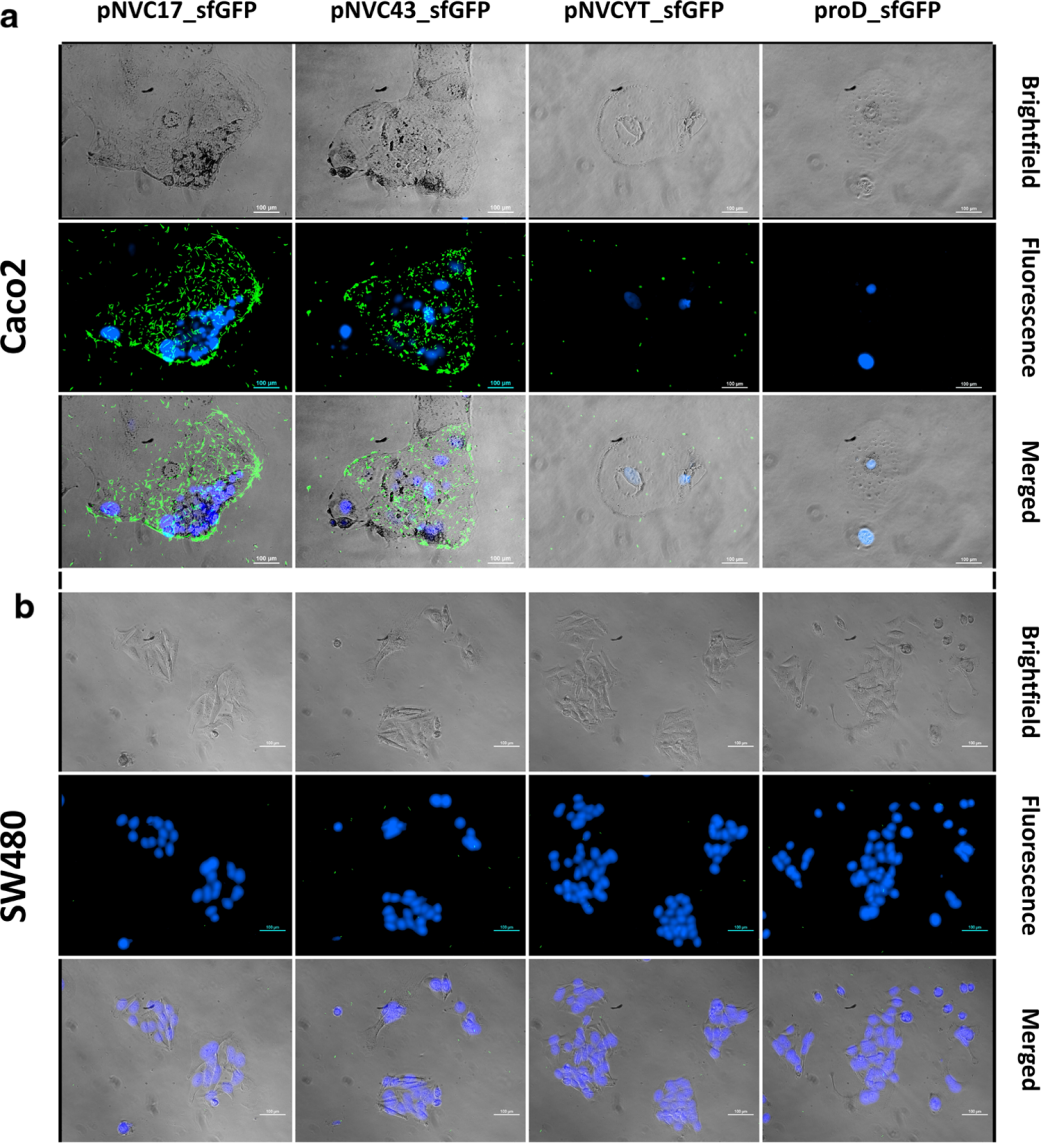
Specific adhesion of engineered *E. coli* to targeted cancer cells after 2 h of incubation. Engineered *E. coli* carrying different pNV plasmids with sfGFP (green) were incubated with two different colorectal cancer cell lines (MOI 300:1): (a) Caco2, a high CEA-expressing cell line and (b) SW480, a low CEA-expressing cell line, in which both are stained with Hoechst dye (blue). prod_sfGFP plasmid is a pNV_sfGFP plasmid without the VHH region (Supporting Information Figure S1). Microscopic images at different time points throughout the 8-h incubation can be found in Supporting Information Figures S1–S3. Scale bar is 100 μm.

For the proof-of-concept experiments, a monolayer of both cancer cell lines, Caco2 or SW80 was incubated with engineered *E. coli* (multiplicity of infection, MOI 300:1) up to 8 h in 24-well plates (Figure 2 and Supporting Information Figures S2–S4). All wells were washed three times with fresh media to reduce nonspecific binding prior to microscopic imaging. All engineered *E. coli* also expressed strong GFP, enabling the visualization of the bacteria on cancer cells. The results show that *E. coli* pNVC17_sfGFP and pNVC43_sfGFP specifically bound to the high CEA-expressing colorectal cancer cell line Caco2 throughout the incubation period (Figure 2 and Supporting Information Figures S2–S4). However, the control strains expressing a non-targeting nanobody (pNVCYT_sfGFP) or without a nanobody (proD_sfGFP) did not bind to Caco2 even with a prolonged incubation of up to 8 h (Figure 2 and Supporting Information Figures S2–S4). None of the engineered *E. coli* strains showed any binding to the low CEA-expressing cell line SW480 (Figure 2 and Supporting Information Figures S2–S4). The results demonstrate that the specific binding of engineered *E. coli* to targeted cancer cells was solely contributed by the nanobodies displayed on the surface.

### Engineering and Purification of Mini-SimCells to Target Colorectal Cancer Cells

**Figure 3:**
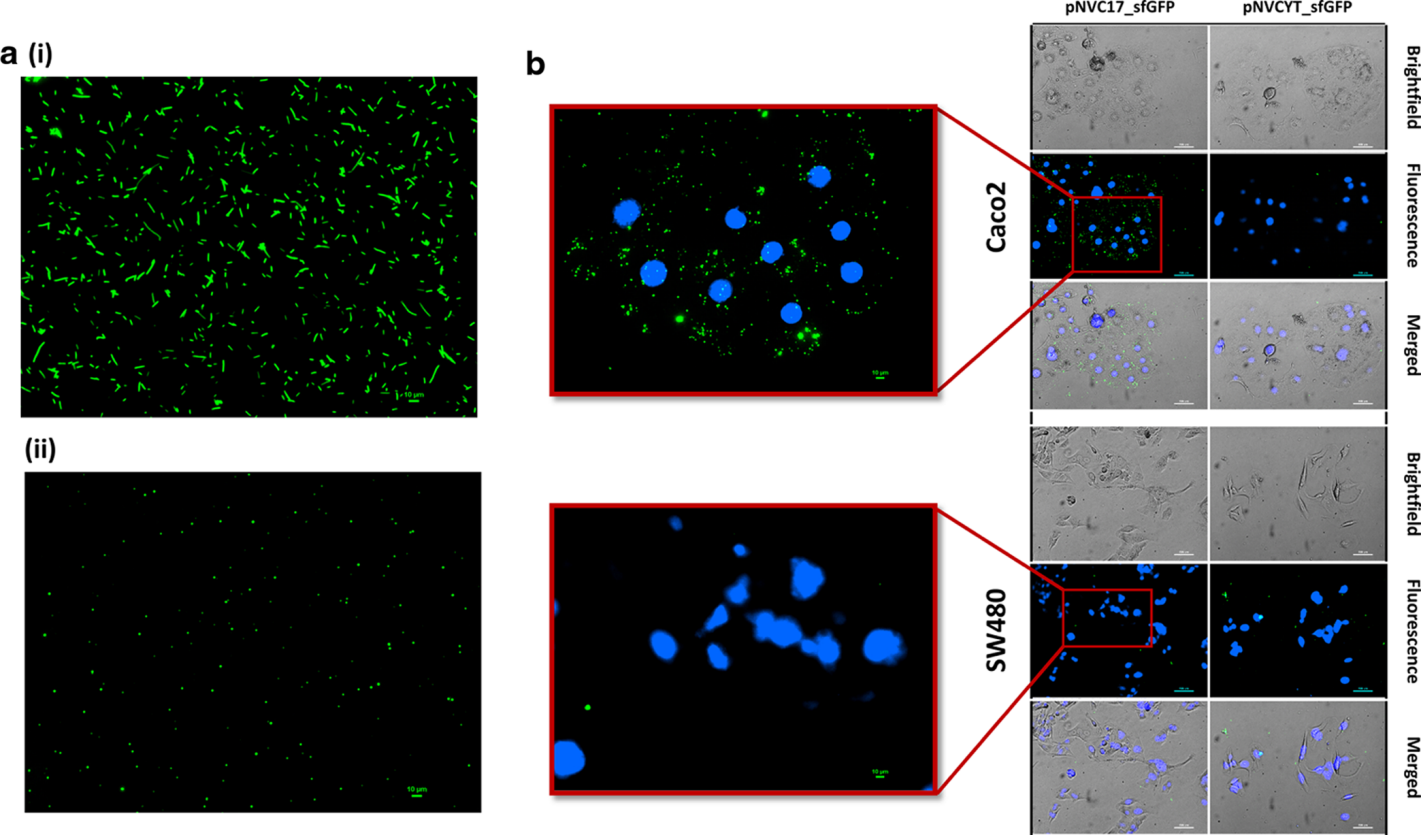
Engineering of *E. coli* mini-SimCells for specific cancer cells adhesion. (a) (i) Pre- and (ii) post-purification of pNVC17_sfGFP transformed *E. coli* mini-SimCells (green). Scale bar is 10 μm. (b) Two-hour incubation of pNVC17_sfGFP or pNVCYT_sfGFP transformed mini-SimCells with high CEA-expressing Caco2 and low CEA-expressing SW480. The red box shows the zoom-in region using 20× magnification. Nuclei were stained with Hoechst dye (blue). Microscopic images at different time points throughout the 8-h incubation can be found in Supporting Information Figure S5. Scale bars are 100 and 10 μm for the zoom-in region.

**Figure 4:**
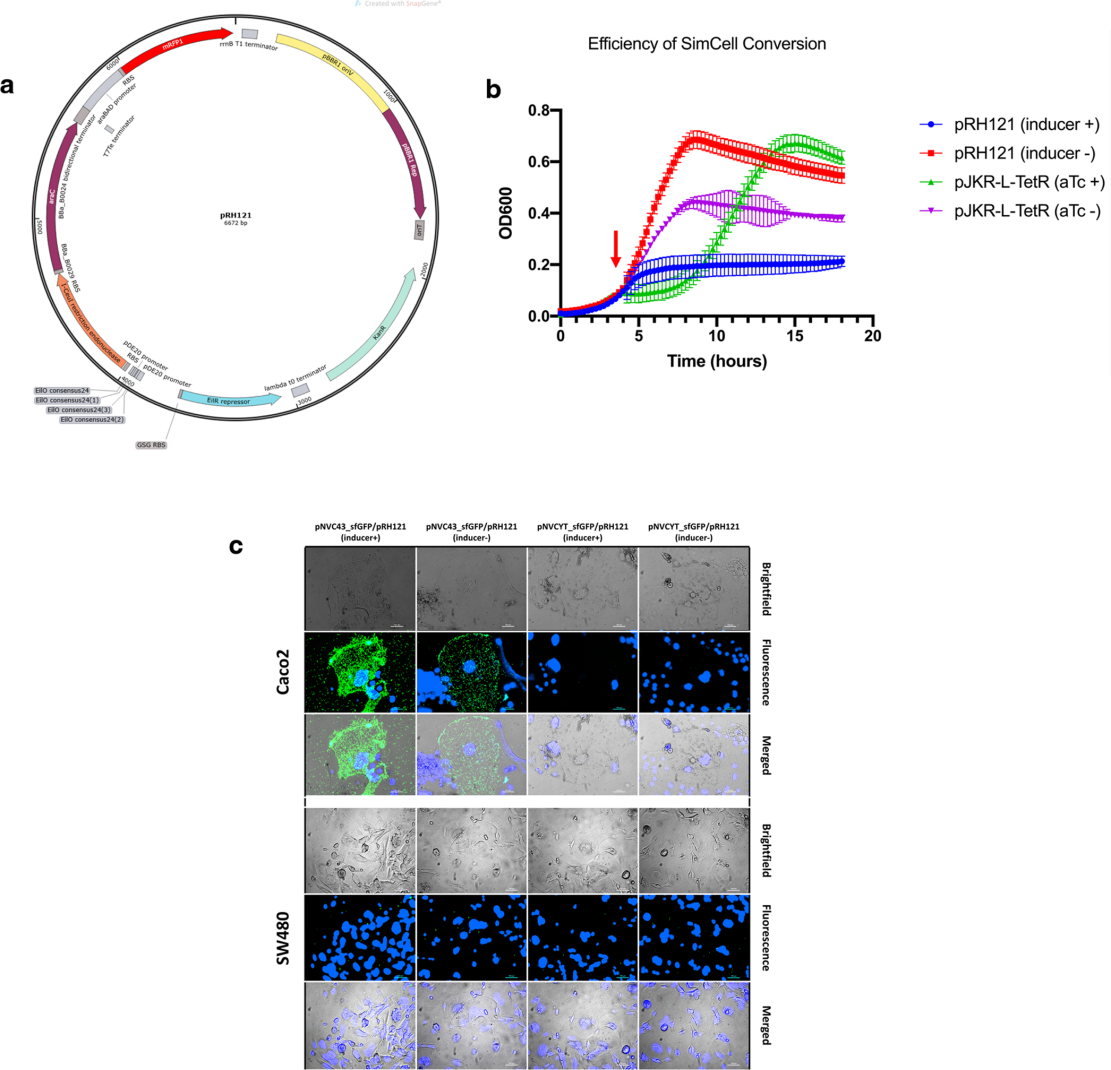
Reprogramming genome-less, nonreplicating SimCells for targeted cancer cell adhesion. (a) Schematic map of pRH121 plasmid. (b) OD_600_ reading of *E. coli* carrying pEH121 and pJKR-L-TetR^22^ (TetR controlled) plasmid for SimCell conversion. The red arrow indicates the time point of the addition of inducer crystal violet (inducer +) and anhydrotetracycline (aTc+) to pRH121 (blue line) and pJKR-L-TetR (green line) culture, respectively. Inducer-(red line) and aTc-(purple line) are the control cultures without the addition of inducer crystal violet and aTc. Error bars represent the standard deviation from three biological repeats. (c) Two-hour incubation of programmed SimCell pRH121 carrying pNVC43_sfGFP or pNVCYT_sfGFP with (+) and without (−) the addition of inducer (green) (MOI 300:1). Bacterial cells under inducer + are genome-less and nondividing SimCells (Figure S6). (i) Caco2 and (ii) SW480 are the high CEA-expressing and low CEA-expressing colorectal cancer cell lines, respectively. Nuclei were stained with Hoechst dye (blue). Microscopic images at different time points throughout the 8-h incubation can be found in Supporting Information Figure S7. Scale bar is 100 μm.

The *minD* gene in *E. coli* plays a crucial role in bacterial cell division by confining the division septum at mid-cell region.^40^ We used a strategy to generate a chromosome-free mini-SimCells by knocking out the *minD* gene. The Δ*minD* mutant *E. coli* will generate minicells due to an aberrant cell division, in which mini-SimCells can then be purified through sequential centrifugation.^41^ We first created the *minD* deleted *E. coli* BL21(DE3) mutant using the Lambda Red recombineering system.^42^ The successful mutant was confirmed via colony PCR (Supporting Information Figure S5a) and observation of mini-SimCell production (Figure 3a). We then transformed the Δ*minD* strain with pNVC17_sfGFP for nanobody surface display. Once the culture reached its late log phase (OD_600_ = 0.6), we removed the parental cells by centrifuging at 2000*g*, followed by the addition of 100 μg/mL of ceftriaxone,^43^ cefotaxime,^44^ and penicillin G.^45^ These antibiotics exerted their bactericidal action through the inhibition of peptidoglycan cell wall synthesis, resulting in the cell lysis of dividing parental cells, while the nondividing mini-SimCells remained intact. This has significantly improved the yield and purity of the mini-SimCells. Figure 4b shows the highly purified mini-SimCells after the purification steps, and the size of mini-SimCells was 100–400 nm. Overnight OD_600_ reading and agar plate incubation of the purified mini-SimCells showed no further growth (Supporting Information Figure S5b,c), indicating that all parent cells have been removed.

To investigate the binding efficacy of engineered mini-SimCells, we repeated the *in vitro* cancer cells binding experiment with mini-SimCells. As shown in Figure 3b and Supporting Information Figure S6, specific binding can only be observed between the high CEA-expressing Caco2 and mini-SimCells engineered with pNVC17_sfGFP. No adhesion was found on the low CEA-expressing SW480 or mini-SimCells transformed with unspecific nanobody pNVCYT_sfGFP. These results indicate that mini-SimCells expressing C17 nanobodies can also specifically bind to cancer cells with CEA.

### Generation and Reprogramming of SimCells to Target Cancer Cells

We recently reported the production and characterization of an I-CeuI endonuclease-induced, chromosome-free SimCell as a programmable synthetic biology chassis.^20^ The generation of SimCell is based upon the recognition of I-CeuI endonuclease on a defined 26-bp sequence ubiquitously found in most bacterial genomes that leads to a RecBCD-initiated double-strand degradation.^46^ We used an optimized SimCell induction plasmid, pRH121^22^ (Figure 4a), to produce SimCells which shows superior performance compared to the TetR controlled pJKR-L-TetR system.^20^ pRH121 showed no visible basal expression, and a complete halt of growth can be achieved within 90 min after the induction, indicating a large amount of SimCell generation from parent cells (Figure 4b).

To reprogram SimCells for cancer cell targeting, we transformed both pRH121 and pNVC43_sfGFP plasmids into *E. coli* BL21(DE3). Once the culture reached its early log phase (OD_600_ = 0.3), we added the inducer to transform the bacterial cells into highly pure, nondividing SimCells culture (see Methods). Overnight agar plate and OD_600_ reading showed no further growth, and no single colony was formed after 72 h of induced SimCells compared to their uninduced counterpart (Supporting Information Figure S7), indicating highly efficient and robust SimCell generation.

We then assessed the binding capacity of programmed SimCells to targeted cancer cells (MOI 300:1). As shown in Figure 4c and Supporting Information Figures S8–S10, adhesion of high CEA-expressing Caco2 was achieved by both induced and uninduced SimCells carrying the pNVC43_sfGFP plasmid within 2 h of incubation. For controls, no binding was observed on the low CEA-expressing SW480. Similar to the live bacteria binding assay, induced and uninduced SimCells carrying the pNVCYT_sfGFP control plasmid did not bind to any of the colorectal cancer cells, which shows the specificity of reprogrammed SimCells to targeted cancer cells.

### Binding of Reprogrammed Mini-SimCells and SimCells Can Induce Targeted Cancer Cell Death In Vitro

Previous attempts to treat cancers using bacterial therapy relied on the colonization and proliferation of bacteria at the tumor site, followed by the expression of different therapeutic payloads such as immunomodulators^8^ and enzymes^17^ to enhance the anticancer property. As mini-SimCells and SimCells were reprogrammed to bind directly onto specific cancer cells, we investigated the cytotoxic effect of this interaction to assess the potential of mini-SimCells and SimCells as stand-alone therapeutics without any additional anticancer circuits.

**Figure 5:**
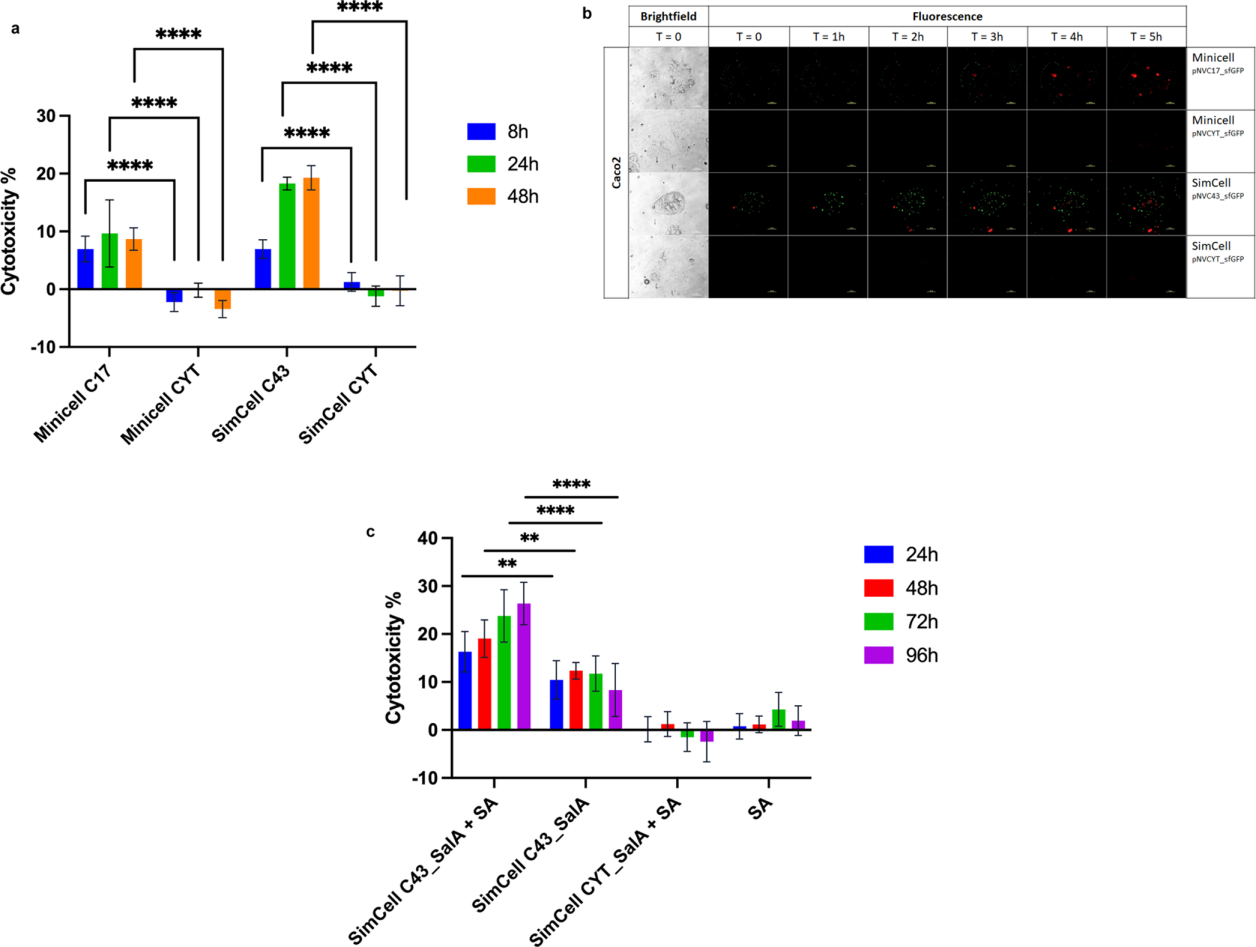
Induction of targeted cancer cell death via SimCell and mini-SimCell *in vitro*. (a) Cytotoxicity of targeted mini-SimCell C17 and SimCell C43 (representing pNVC17_sfGFP and pNVC43_sfGFP, respectively) toward Caco2 compared to their nonspecific counterparts CYT (representing pNVCYT_sfGFP). Caco2 was first incubated with mini-SimCells and SimCells for 2 h, followed by washing thrice with fresh media prior to further incubation at 37 °C with 5% CO_2_. LDH assay was used to measure cytotoxicity at *T* = 8, 24, and 48 h. Error bars represent the standard deviation from eight biological repeats. (b) Time-lapse images of Caco2 at time point 0, 1, 2, 3, 4, and 5 h after 2 h of incubation with mini-SimCell (pNVC17_sfGFP and pNVCYT_sfGFP) and SimCell (pNVC43_sfGFP and pNVCYT_sfGFP). All cultures were washed thrice and added with fresh media supplemented with ethidium homodimer prior to imaging. The remaining SimCells and mini-SimCells are in green and the nuclei of Caco2 with compromised membrane were stained by Ethidium homodimer (red). Scale bar is 100 μm. The full 5-h time-lapse video can be found in Supporting Information Movie S1. (c) Cytotoxicity of targeted SimCell C43_SalA (representing pNVC43_SalA_sfGFP) toward Caco2 with and without the addition of salicylic acid (SA) in comparison to preincubation with nonspecific SimCell CYT_SalA (representing pNVCYT_SalA_sfGFP). Caco2 was first incubated with SimCells for 2 h, followed by washing thrice with fresh media supplemented with 500 μM of SA prior to further incubation at 37 °C with 5% CO_2_. LDH assay was used to measure the cytotoxicity at *T* = 24, 48, 72, and 96 h. Error bars represent the standard deviation from eight biological repeats. Statistical test performed is 2-way ANOVA, **p ≤ 0.01; and ****p ≤ 0.0001.

We first incubated Caco2 with mini-SimCells and SimCells for 2 h, followed by washing thrice to remove any unbound bacteria. The Caco2 culture was then incubated at ideal conditions until a specific time point to run the cytotoxicity assay. As shown in Figure 5a, mini-SimCells and SimCells carrying pNVC17_sfGFP and pNVC43_sfGFP, respectively, can induce cancer cell killing effect from 8 h of incubation after the removal of unbound SimCells, with a higher cytotoxicity effect observed in SimCell at 24 h (18.3% cancer cell damage) and 48 h (19.3% cancer cell damage) of incubation. In contrast, no significant cytotoxicity was observed in nonspecific mini-SimCells and SimCells (pNVCYT_sfGFP) post-washing despite the initial 2-h incubation. Despite the relatively low cytotoxicity effect, this result suggests that the specific binding of mini-SimCells and SimCells can exert their cancer-killing effect gradually without any additional active cancer-killing circuits. According to the fluorescent images of SimCells and mini-SimCells on Caco2 cells, we estimate that there were about 474 ± 45 SimCells and 623 ± 197 mini-SimCells bound to a Caco2 cell. The difference might be due to the different nanobodies of C17 displayed on mini-SimCells and C43 on SimCells.

To further examine the killing mechanism, we incubated the Caco2 cells with mini-SimCells and SimCells, as well as ethidium homodimer, a membrane-impermeable nucleic acid dye that is commonly used for dead-cell staining.^47^ The cell mixtures were monitored using a time-lapse fluorescence microscopy after 2 h of incubation, followed by triple washing to remove unbound SimCells and mini-SimCells (Figure 5b and Supporting Information Movie S1). Caco2 bound by targeted mini-SimCells (pNVC17_sfGFP) and SimCells (pNVC43_sfGFP) starts showing red fluorescence from 2 h of microscopic imaging post-washing, indicating a compromised cancer cell membrane due to mini-SimCells and SimCells binding. In contrast, incubation with nonspecific mini-SimCells and SimCells does not generate significant membrane damage on the cancer cell within 5 h post-washing, which confirms that the cytotoxicity effect is exerted by specific binding of mini-SimCells and SimCells. As shown at *T* = 5 h, mini-SimCells and SimCells are unbound from the dead cancer cells and dissipate to their surrounding. This result potentially reflects the gradual cell-damaging effect seen in Figure 5a, in which the increase in cytotoxicity from 8 to 24 h of incubation could be a result of the bystander effect caused by mini-SimCells and SimCells binding from one cancer cell to another. However, it is important to note that microscopic imaging was carried out in less than ideal conditions (no temperature and CO_2_ control), which accounts for the shorter time scale (5 h in the microscopy experiment compared to 8 h in the cytotoxicity assay) for cancer cell killing.

SimCells can also be engineered to carry “cargo” gene circuits to produce anticancer compounds. As a proof of concept to enhance its anticancer property, we cloned *salA* gene encoding for salicylate hydroxylase,^20,48^ upstream of *sfGFP* in the pNVC43_sfGFP plasmid. SalA and indigenous esterase of *E. coli* can convert salicylate/aspirin into catechol, which has been previously shown to exert apoptotic effect toward selective cancer cell lines.^20,48−50^ Using a similar protocol, we built SimCells pNVC43_SalA_sfGFP/pRH121 and repeated the *in vitro* cancer-killing experiments with the addition of 500 μM salicylic acid, which are within the safe range of the aspirin/salicylate dosage.^29^ As shown in Figure 5c, the addition of salicylic acid enhanced the cancer-killing effect of engineered SimCells compared to SimCells binding alone. No significant cytotoxic effect was observed in cancer cell cultures that contained only salicylic acid, including when it was preincubated with SimCells pNVCYT_SalA_sfGFP for 2 h. Despite the fact that further improvement will be necessary for its clinical relevance, these results demonstrate the therapeutic potential of targeted mini-SimCells and SimCells *in vitro*. The further improvement of these chassis of SimCells and mini-SimCells will possess enormous advantages over conventional bacterial therapy with their high specificity and biological safety.

## Discussion

An important lesson from the past clinical trials using living bacteria for cancer treatment is that their intrinsic ability to colonize the tumor microenvironment does not always translate from animal models to humans (reviewed in^4^). This gives rise to another important question: in the cases where tumor regression was observed, is the colonization and proliferation of bacteria at the tumor microenvironment accountable for the therapeutic effect, or is it a mere bacteria-incited cytokine storm? To realize the potential of bacterial cancer therapy, it is desirable that the engineered bacteria can specifically target cancer cells, delivering killing agents and recruiting T cells *in vivo*. In addition, one of the biggest hurdles to translating living therapeutics for clinical use is the regulatory considerations in deploying replication-competent bacteria in patients.^4^ In this study, we addressed both concerns by engineering the nonreplicating synthetic biology chassis, mini-SimCells, and SimCells, to develop a targeted cancer therapeutic. Mini-SimCells (100–400 nm) are smaller than normal bacteria (1–2 μm), able to penetrate deeply into the tumor. Mini-SimCells usually require genome engineering and are only applicable to those bacteria containing minCD genes. SimCells, in theory, can be generated from any bacteria as engineered I-CeuI recognizes and cuts 26-base pair sequence (TAACTATAACGGTCCTAAGGTAGCGA) within conserved *rrl* gene of 23S rRNA (present in most bacterial chromosome), creating multiple double-stranded breaks and leading to the chromosome degradation by RecBCD nuclease.^22^ Nondividing mini-SimCells and SimCells are able to synthesize and deliver toxic compounds (e.g., catechol),^22^ which would kill wild-type living bacteria. For example, it has been demonstrated that minicells (chassis of mini-SimCells) can be loaded to release super-cytotoxic anti-tumor drug.^51^

We first developed a CEA diagnostic system using engineered *E. coli* surface-displaying anti-CEA nanobodies in a biological agglutination test setting. In contrast to a conventional bacterial biosensing system that requires active production of signal protein such as GFP or Lux protein, this simple platform allows the detection of a cancer biomarker through the physical binding between engineered bacteria and target analyte. Further modulation on the sensitivity of the test is possible through the tuning of expression strength of anti-CEA nanobody on the bacterial surface. Previous reports also showed the usability of the biological agglutination test on more complex samples such as urine and blood.^35,36^ Ideally, the CEA diagnostic system should be built to reach a sensitivity of 2.2 ng/mL (∼12 pM) to have clinical significance.^52^

We showed the modularity of the pNV system in conferring cell binding specificity to engineered bacteria. With simple molecular cloning steps, we could modify the nanobody sequence to expand the cell-binding repertoire. As shown in the *in vitro* cancer cell binding experiment, anti-CEA nanobodies C17 and C43 can be used interchangeably without affecting the binding capacity of engineered bacteria. To further enhance the binding strength or to identify a new target of interest, we could adopt a phage surface display library or induce an immune response in Llama to generate new nanobody variants.^53,54^

Recent advances in synthetic biology have promised controllable, tumor-specific, and user-defined therapeutic payloads for bacterial cancer therapy (reviewed in^1,2,4,7,26^). While the collection of synthetic biology circuits (such as quorum lysis^55^ and genetic kill switches^56^) for *in vivo* therapeutic applications continues to grow, the genetic stability and spreading of living therapeutics within a patient remain one of the biggest concerns to regulatory bodies.^26,57^ With mini-SimCells and SimCells, we remove the growth aspect of the living bacteria and thereby the inherent risk of a gene mutation that comes with every doubling cycle. We showed the capability of both mini-SimCells and SimCells as stand-alone anticancer agents through *in vitro* experiments and by combining a user-defined therapeutic circuit, in this case, the *salA* gene and salicylic acid, SimCells can exert a higher cytotoxic effect to achieve targeted cancer-killing. As the binding and cancer-killing mechanism can be delivered through independent genetic circuits, these chassis can be further customized and expanded to target other diseases. The modularity, scalability, and reliability of these chassis can accentuate the impact of synthetic biology in the medical field. We envision that the deployment of SimCell therapy will open a new frontier of cancer treatment.

## Methods

### Bacterial Strains, Plasmids, Primers, and Routine Cell Growth Conditions

A list of bacterial strains and plasmids used in this study is provided in Table S1. In general, *E. coli* DH5α was used for routine cloning and plasmid maintenance, while *E. coli* BL21(DE3) was used for the rest of the study unless stated otherwise.

Primers and gene blocks used in this study are listed in Table S2. All primers and gene blocks were ordered from Sigma-Aldrich. Parts and backbones were amplified using Q5 High Fidelity DNA Polymerase (New England Biolabs, NEB), and plasmids were constructed using NEBuilder Hifi DNA Assembly (NEB).

For all routine bacterial cell growth and strain selection, bacteria were grown in Luria–Bertani (LB) media (with agitation, 250 rpm) or plated on LB agar (static) with corresponding antibiotics: kanamycin (Kan, 50 μg/mL), carbenicillin (Carb, 100 μg/mL), or chloramphenicol (Cm, 25 μg/mL) and incubated at 37 °C, for 16 h.

All strains were transformed via heat shock unless stated otherwise. Chemically competent cells and transformation were conducted according to methods described previously.^58,59^ For strains containing two plasmids, the transformation was done sequentially by first making the single-plasmid strain chemically competent again.

### Cancer Cell Lines and Routine Cell Growth Conditions

Human colorectal cancer cell lines Caco2 and SW480 were kindly gifted by Professor Adrian Harris from the Department of Oncology, University of Oxford. Both cell lines were routinely grown as a monolayer in fresh media (Dulbecco’s modified Eagle’s medium (DMEM, high glucose, pyruvate) supplemented with 10% fetal bovine serum (FBS) and Penicillin–Streptomycin solution (100 U/mL)) at 37 °C with 5% CO_2_. Cells were generally grown to 70% confluency before sub-culturing or transfer to a 24-well plate or a 96-well plate.

### Quantification of Bacterial Surface Expressing Nanobody Using Flow Cytometry

Overnight BL21(DE3) culture carrying pNV_2 or pNV_3 plasmid (5 mL) was centrifuged at 4 °C, 2000*g* for 5 min. Briefly, 1 mL of nonfat milk blocking buffer (1%) in PBS was added to resuspend the cell pellet. The culture was incubated at room temperature for 1 h. Then, 1.5 mL of the culture was transferred to an Eppendorf tube and was centrifuged at 4000*g* for 2 min. The pellet was washed twice and resuspended in 1 mL of PBS. Then, 2 μL of primary anti-Myc antibody (Abcam, ab9106) was added to the 1 mL cell suspension and incubated at room temperature for 1.5 h. The suspension was centrifuged at 1000*g* for 5 min and washed twice with 1 mL of PBS. The pellet was resuspended in 500 μL of PBS and added with 0.5 μL of secondary Alexa Fluor 488 conjugated antibody (Abcam, ab150077). The culture was incubated at room temperature for 1 h. Finally, the culture was centrifuged at 1000*g* for 5 min, washed twice, and resuspended in 1 mL of PBS. Flow cytometry was done using a FACS Calibur (BD Biosciences) and analyzed using CellQuest. The FL1 filter used to detect Alexa Fluor 488 has an excitation/emission at wavelength 488/530 nm.

### Biological Agglutination Test

Overnight culture carrying pNV_2_sfGFP or pNV_3_sfGFP plasmid was diluted in PBS to OD_600_ = 0.5. Briefly, 100 μL of the diluted culture was transferred to a clear, round-bottom 96-well plate, supplemented with a series of concentrations of human CEACAM5 protein (SinoBiological) ranging from 1 to 200 nM (volume added 0.5–2.5 μL). The plate was incubated statically at room temperature overnight before a top-view image was taken with VersaDoc Imaging system (Bio-Rad) under FITC channel.

### Generation of BL21(DE3) ΔminD

Kanamycin resistance gene cassette (Kan^R^ cassette) was amplified using Q5 High Fidelity DNA Polymerase (NEB) using primers minD-HR-Kan-for and minD-HR-Kan-rev, followed by PCR purification using QIAquick PCR purification kit (QIAGEN).

All cloning and plasmid maintenance of pSIJ8 were done at 30 °C due to its temperature sensitivity. Overnight BL21(DE3) culture transformed with pSIJ8 was sub-cultured 1:100 in 150 mL fresh LB-Carb media and incubated at 30 °C, 250 rpm for 3 h. Arabinose (15 mM) was added to induce the recombination machinery (*exo*, *bet*, and *gam*) and incubated for further 45 min at 30 °C, 250 rpm. The bacterial culture was then made electrocompetent following the methods described previously.^60^

Kan^R^ cassette was transformed into BL21 pSIJ8 via electroporation: 50 uL of electrocompetent BL21 pSIJ8 was added with ∼250 ng Kan^R^ cassette (<2 μL) and mixed by pipetting up and down. The mixture was transferred into a 0.2 cm electroporation cuvette (Bio-Rad) and pulsed at 2.5 kV using Cellject Uno (Thermo Fisher Scientific). Then, 1 mL of fresh LB was added immediately into the cuvette and mixed by pipetting up and down. The mixture was transferred to an Eppendorf tube and incubated at 30 °C, 250 rpm for 4 h. The mixture was centrifuged at 4500*g* for 10 min, and the supernatant was removed until ∼100 μL was left in the Eppendorf tube. Cell pellet was resuspended in the leftover media and plated on LB-Carb-Kan agar at 30 °C, overnight. On the next day, colony PCR was done using primers HR-check-for and HR-check-rev for success recombination. The colony was inoculated in LB-Kan and incubated at 37 °C, 250 rpm overnight to cure the pSIJ8 plasmid and make BL21 Δ*minD::Kan*^*R*^ culture. This culture was subsequently made chemically competent to be transformed with the pNV plasmids.

### Purification of Mini-SimCells

Overnight BL21 Δ*minD* culture was diluted 1:100 in 300 mL of LB-Kan-Cm and incubated at 37 °C, 250 rpm for 3–4 h until OD_600_ = 0.6. The culture was centrifuged at 2000*g* for 10 min at 4 °C, and the supernatant was retained for further centrifugation at 12,000*g* for 15 min at 4 °C. The pellet was resuspended in 1 mL of fresh LB, pooled together and incubated at 37 °C, 250 rpm for 45 min. Then, ceftriaxone (100 μg/mL), penicillin G (100 μg/mL), and cefotaxime (100 μg/mL) were added to the culture and further incubated at 37 °C, 250 rpm for 2 h. The culture was first centrifuged at 500 g for 10 min at 4 °C to remove cell debris. The supernatant was retained and further centrifuged at 12,000*g* for 15 min at 4 °C. The final pellet was resuspended in 1 mL of PBS and stored at 4 °C until further use. This purified culture is the mini-SimCells.

Then, 5 μL of prepurified and post-purified culture were plated on LB-Cm-Kan agar plate and added into 195 μL of LB-Cm-Kan media in a 96-well plate, incubated overnight at 37 °C to collect OD_600_ readings.

### Generation of Genome-Less SimCells

To characterize SimCell conversion, *E. coli* K12 MDS42 was transformed with pJKR-L-TetR-I-CeuI (TetR controlled system) and compared to SimCell pRH121.^22^ Overnight cultures were diluted 1:100 in 200 μL of LB-Kan and transferred into a flat 96-well plate. The plate was sealed with Breathe-Easy sealing membrane and was incubated in the Synergy 2 microplate reader (BioTek) at 37 °C, with constant orbital shaking at 1000 rpm. OD_600_ measurements were taken every 15 min for 18 h. At the 4th hour, 0.2 μL of inducer crystal violet or anhydrotetracycline (final concentration 1 μM) was added into *E. coli* with pRH121 or pJKR-L-TetR-I-CeuI culture, respectively.

To reprogram SimCells to surface display nanobody, overnight culture of *E. coli* BL21 containing pRH121^22^ and pNVC43_sfGFP or pNVCYT_sfGFP plasmid was diluted 1:100 in LB-Cm-Kan (50 mL) and incubated at 37 °C, 250 rpm for 3 h until an OD_600_ of ∼0.3. The culture was split into half: 25 mL of “Uninduced” culture was rested at 4 °C until further use; 25 mL of “Induced” culture was added with crystal violet to induce the conversion of SimCells. Induced culture was incubated at 37 °C, 250 rpm for another 2 h before storing at 4 °C until further use. This Induced culture is the genome-less SimCells.

Briefly, 5 μL of uninduced and induced cultures were plated on LB-Cm-Kan agar plate and added into 195 μL of LB-Cm-Kan media in a 96-well plate and incubated overnight at 37 °C to collect OD_600_ readings using a plate reader (BioTek Synergy 2).

#### *In Vitro* Cancer Binding

Cancer cells were seeded in a 24-well tissue culture plate (∼50,000 cells/well) with 0.5 mL fresh media and grown at 37 °C with 5% CO_2_ to 70% confluency.

For *E. coli* BL21(DE3) strains, overnight bacteria culture was diluted to OD_600_ = 0.3 (∼3 × 10^7^ CFU/mL by plating on LB agar with the corresponding antibiotic) in PBS and centrifuged at 5000*g* for 5 min. After removing the supernatant, the cell pellet was resuspended in fresh media supplemented with Hoechst dye (0.5 μM) at the same volume (i.e., 10 mL diluted bacteria culture in PBS resuspended into 10 mL of supplemented media after centrifugation and supernatant removal). For SimCell strains, Induced culture was centrifuged at 5000*g* for 5 min, followed by similar steps as the BL21(DE3) strains. For mini-SimCell strains, Hoechst dye (0.5 μM) was added into purified mini-SimCells in PBS directly.

Media for cancer cell culture in the 24-well plate was removed and washed once with fresh media. For an infection at MOI of 300:1, 0.5 mL of BL21(DE3), SimCells or mini-SimCells were added into each well for incubation at 37 °C with 5% CO_2_ for 0, 2, 4, and 8 h. For time point 0, 0.5 mL fresh media supplemented with Hoechst dye (0.5 μM) was added and incubated for 15 min prior to the addition of bacteria. At each time point, each well was washed thrice and added with 0.5 mL of fresh media before subjecting to fluorescence microscope imaging under 10x magnification using Eclipse Ti fluorescence microscope (Nikon). The fields were viewed under brightfield or fluorescent illuminance (excitation/emission for sfGFP: 488/510 nm; Hoechst dye: 361/497 nm) at 10× or 20× objective.

#### *In Vitro* Cancer-Killing

For plate-reading experiment, Caco2 cells were first seeded in a 96-well tissue culture plate (∼10,000 cells/well) with 100 μL of fresh media and grown at 37 °C with 5% CO_2_ to 70% confluency. For an infection at MOI of 300:1, mini-SimCells and SimCells were prepared as described in the previous section, and 100 μL was added into each well for incubation at 37 °C with 5% CO_2_ for 2 h. After 2 h of incubation, each well was washed thrice and added with 100 μL of fresh media, followed by further incubation at 37 °C with 5% CO_2_. For experiments using SimCells pNVC43_SalA_sfGFP, 100 μL of fresh media supplemented with salicylic acid (final concentration 500 μM) was added after the washing step. At *T* = 8, 24, and 48 h (and *T* = 72 and 96 h for experiments using SimCells pNVC43_SalA_sfGFP), cytotoxicity assay was carried out using LDH Cytotoxicity Assay Kit (Abcam) following the manufacturer’s instructions. Briefly, 5 μL of supernatant from each well was added into 95 μL of LDH reaction mix in another white, flat-bottom 96-well plate and shaken gently at room temperature for 10 min. Fluorescence (excitation/emission: 535/587 nm) was measured using a plate reader (SpectraMax i3x). The percentage cytotoxicity is calculated using the equation given below
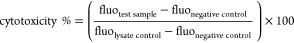


Cell culture was returned to 37 °C, 5% CO_2_ incubation after each time point. For time-lapse experiment, Caco2 cells were first seeded in 24-well tissue culture plate (∼50,000 cells/well) with 0.5 mL of fresh media and grown at 37 °C with 5% CO_2_ to 70% confluency. Mini-SimCells and SimCells were prepared as described in the previous section, and 0.5 mL was added into each well for incubation at 37 °C with 5% CO_2_ for 2 h. After 2 h of incubation, each well was washed thrice and added with 0.5 mL of fresh media supplemented with ethidium homodimer (4 μM). The cultures were subjected to fluorescence microscope imaging under 10× magnification using Eclipse Ti fluorescence microscope (Nikon) for 5 h with an interval of 5 min. The fields were viewed under brightfield for *T* = 0 and fluorescent illuminance (excitation/emission for sfGFP: 488/510 nm; ethidium homodimer: 528/617 nm) for the time-lapse.

## Supporting Information Available

Contains further details about plasmid and bacterial strain constructions, supplemental tables, references, and figures (PDF)
Time-lapse video of Caco2 after 2 h of incubation with SimCell + pNVC43_sfGFP (top left); SimCell + pNVCYT_sfGFP (bottom left); minicell + pNVC17_sfGFP (top right); and minicell + pNVCYT_sfGFP (bottom right). All cultures were washed thrice and added with fresh media supplemented with ethidium homodimer prior to imaging. The duration of the experiment is labeled in hh:mm:ss format on the top left. SimCell and minicell are in green and the nuclei of Caco2 with compromised membrane were stained by ethidium homodimer (red). Scale bar is 100 μm (Movie S1) (MP4)

## Notes

All data discussed in the paper will be made available to readers.
