# Supplementary material for: Reprogramming Synthetic Cells for Targeted Cancer Therapy
Source: ACS Synth Biol. 2022 Mar 8;11(3):1349–60. doi: 10.1021/acssynbio.1c00631 (PMC9084601; doi:10.1021/acssynbio.1c00631)
Supplement: Supplementary file 1 — sb1c00631_si_001.pdf [file sb1c00631_si_001.pdf]

Supplementary information

Reprogramming Synthetic Cells for Targeted Cancer Therapy

Boon Lim[1,2#], Yutong Yin[1#], Hua Ye[1,2], Zhanfeng Cui[1,2], Antonis Papachristodoulou[1], Wei E Huang[1,*]

[1]Department of Engineering Science, University of Oxford, Parks Road, OX1 3PJ, Oxford, UK.

[2]Institute of Biomedical Engineering, Department of Engineering Science, University of Oxford, OX3 7DQ, UK.

[*]Corresponding author: Department of Engineering Science, University of Oxford, Parks Road, OX1 3PJ, Oxford, United Kingdom. Email wei.huang@eng.ox.ac.uk Tel: +44 1865 283786.

[#] B.L. and Y.Y contributed equally to this work.

Table S1. Bacterial strains and plasmids used in this study.

Strains	Genotype or description	Source
Escherichia coli DH5α	*fhuA2 lac(del)U169 phoA glnV44 Φ80' lacZ(del)M15 gyrA96 recA1 relA1 endA1 thi-1 hsdR17*	Lab collection
E. coli BL21(DE3)	*fhuA2 [lon] ompT gal (λ DE3) [dcm] ΔhsdS*	Lab collection
E. coli K12 MDS42	*fhuACDB(del) endA(del)* and deletion of all IS and cryptic prophage[1]	Lab collection
DH5α pNVC17	BL21(DE3): pNVC17 (for cloning)	This study
BL21(DE3) pNVC17_2	BL21(DE3): pNVC17_2 (low expression profile)	This study
BL21(DE3) pNVC43_2	BL21(DE3): pNVC43_2 (low expression profile)	This study
BL21(DE3) pNVCYT_2	BL21(DE3): pNVCYT_2 (low expression profile)	This study
BL21(DE3) pNVC17_3	BL21(DE3): pNVC17_3 (high expression profile)	This study
BL21(DE3) pNVC43_3	BL21(DE3): pNVC43_3 (high expression profile)	This study
BL21(DE3) pNVCYT_3	BL21(DE3): pNVCYT_3 (high expression profile)	This study
BL21(DE3) control	BL21(DE3): proD_sfGFP (high expression profile control with sfGFP)	This study
BL21(DE3) pNVC17_sfGFP	BL21(DE3): pNVC17_sfGFP (high expression profile with sfGFP downstream)	This study
BL21(DE3) pNVC43_sfGFP	BL21(DE3): pNVC43_sfGFP (high expression profile with sfGFP downstream)	This study
BL21(DE3) pNVCYT_sfGFP	BL21(DE3): pNVCYT_sfGFP (high expression profile with sfGFP downstream)	This study
BL21(DE3) pNVC17_sfGFP_ICeuI	BL21(DE3): pNVC17_sfGFP + pRH12x (high expression profile with sfGFP downstream; ICeuI endonuclease expression)	This study
BL21(DE3) pNVC43_sfGFP_ICeuI	BL21(DE3): pNVC43_sfGFP + pRH12x (high expression profile with sfGFP downstream; ICeuI endonuclease expression)	This study
BL21(DE3) pNVC43_SalA_sfGFP _ICeuI_	BL21(DE3): pNVC43_salA_sfGFP + pRH12x (high expression profile with sfGFP downstream; ICeuI endonuclease expression)	This study
BL21(DE3) pNVCYT_sfGFP_ICeuI	BL21(DE3): pNVCYT_sfGFP + pRH12x (high expression profile with sfGFP downstream; ICeuI endonuclease expression)	This study
BL21(DE3) pSIJ8	BL21(DE3): pSIJ8 (for recombination engineering)	This study
BL21(DE3) Δ*minD*	BL21(DE3) Δ*minD::Kan*^{R} (for minicells generation)	This study
BL21(DE3) Δ*minD* pNVC17_sfGFP	BL21(DE3): pNVC17_sfGFP, Δ*minD::Kan*^{R} (for minicells with high expression profile with sfGFP downstream)	This study
BL21(DE3) Δ*minD* pNVC43_sfGFP	BL21(DE3): pNVC43_sfGFP, Δ*minD::Kan*^{R} (for minicells with high expression profile with sfGFP downstream)	This study

BL21(DE3) Δ*minD* pNVCYT_sfGFP	BL21(DE3): pNVCYT_sfGFP, Δ*minD::Kan*^{R} (for minicells with high expression profile with sfGFP downstream)	This study
**Plasmids**		
pNVgfp	Plasmid with anti-GFP nanobody attached to β-intimin, constitutively expressed by the promoter BBa_J23106 and a low strength RBS (12K TIR). Cm^{R}.	A gift from Dr Karen Polizzi[2]
pNVC17	Anti-GFP nanobody of pNVgfp replaced by anti-CEA nanobody. Cm^{R}.	This study
pNVC43	Anti-GFP nanobody of pNVgfp replaced by another variant of anti-CEA nanobody. Cm^{R}.	This study
pNVC17_2	BBa_J23106 of pNVC17 replaced by BBa_J23105. Cm^{R}.	This study
pNVC43_2	BBa_J23106 of pNVC43 replaced by BBa_J23105. Cm^{R}.	This study
pNVCYT_2	Anti-CEA nanobody of pNVC17_2 replaced by anti-spike protein nanobody. Cm^{R}.	This study
pNVC17_3	BBa_J23106 and RBS of pNVC17 replaced by BBa_K1741014 and BBa_K1758100, respectively.	This study
pNVC43_3	Anti-CEA nanobody of pNVC17_3 replaced by another anti-CEA nanobody variant. Cm^{R}.	This study
pNVCYT_3	Anti-CEA nanobody of pNVC17_3 replaced by anti-spike protein nanobody. Cm^{R}.	This study
pNVC17_sfGFP	BBa_K1758100 and *sfGFP* cloned downstream to the anti-CEA nanobody in pNVC17_3. Cm^{R}.	This study
pNVC43_ sfGFP	Anti-CEA nanobody of pNVC17_sfGFP replaced by another anti-CEA nanobody variant. Cm^{R}.	This study
pNVC43_SalA_sfGFP	*salA* cloned upstream of *sfGFP* within pNVC43_sfGFP. Cm^{R}.	This study
pNVCYT_ sfGFP	Anti-CEA nanobody of pNVC17_sfGFP replaced by anti-spike protein nanobody. Cm^{R}.	This study
prod_sfGFP	A lab plasmid with BBa_K1741014 and BBa_K1758100 upstream of a *sfGFP*. Cm^{R}.	Lab collection
pJKR-L-TetR-ICeuI	Plasmid with I-CeuI endonuclease controlled by TetR. Carb^{R}.	[3]
pRH12x	Plasmid with ICeuI endonuclease controlled by a crystal violet inducible promoter. Kan^{R}.	This study
pSIJ8	Plasmid with lambda Red recombineering genes (*exo*, *bet* and *gam*) and a flippase (FLP) recombinase. Carb^{R}.	[4]

Table S2. Primers and gene blocks used in this study.

**Primers**	**Sequence (5' → 3')**	**Part/Purpose**	**Plasmid**
VHH-for	GAACCGGCCCAGCCGGCCGAAGTTCAACTGGTTGAAAG	Anti-CEA nanobody	pNVC17, pNVC43, pNVC17_2, pNVC43_2
VHH-rev	GATGAGTTTTTGTTCGGCGGCCGCGCTGCTAACTGTAACCTG		

pNV-for	GCTCGAGCCGAACAAAAACTCATCTCAGAAGAGGATGCAGCTGC	pNVgfp backbone	pNVC17, pNVC43, pNVC17_2, pNVC43_2, pNVCYT_2
pNV-rev	GGCCGGCTGGGCCGGTTCCAGC		
pNV-3-for	ATGATTACTCATGGTTGTTATACCCGG	pNVC17 backbone	pNVC17_3, pNVC43_3, pNVCYT_3
pNV-3-rev	GCACACGGTCACACTGCTTC		
pNV-sfGFP-for	GCATGGATGAGCTCTACAAAAAGCTTGACCTGTGAAGTG	pNVC17_3 backbone	pNVC17_sfGFP, pNVC43_sfGFP, pNVCYT_sfGFP
pNV-sfGFP-rev	ATGGTACCTTTCTCCTCTTTTTATGCAGCTGCATCCTC		
sfGFP-for	AAAGAGGAGAAAGGTACCATGAGCAAAG	sfGFP	pNVC17_sfGFP, pNVC43_sfGFP, pNVCYT_sfGFP
sfGFP-rev	TTTGTAGAGCTCATCCATGCCATGTG		
minD-HR-Kan-for	GTTGGCTGTGTTTTTCTTCCGCGAGAGAAAGAAATCGAGTAATGCCATAACTTAGAAAAACTCATCGAGCATCAAATG	Kanamycin resistance gene cassette	-
minD-HR-Kan-rev	CGCTTTGACCGTTCAACCGTTAAATTGATCCCTTTTTAACAAGGAATTTCTATGAGCCATATTCAACGGGAAAC		-
HR-check-for	GACCTCAAGAATATCTTTACGCAACTG	Check for success recombination	-
HR-check-rev	CTGTTTGTAATTGTCCTTTTAACAGCG		-
**Gene blocks**			
VHH-C17	GAAGTTCAACTGGTTGAAAGCGGTGGTGGTTTTGTTCAGGCAGGCGAAAGTCTGACCCTGAGCTGTACCAGCAGCACCCTGACCTTTACACCGTATCGTATGGCATGGTATCGTCAGGCACCGGGTAAACAGCGTGATCTGGTTGCAGATATTAGCAGCGGTGATGGTCGTACCACCAATTATGCAGATTTTGCCAAAGGTCGTTTTACCATTAGCCGTGACAATATCAAAAACACCGTTTTTCTGCGTATGACCAATCTGAAACCGGAAGATACCGCAGTGTATTATTGTAATACCTTTGTGAGCTTTGTGGGTATTGCACGTAGCTGGGGTCAGGGCACCCAGGTTACAGTTAGCAGC	Anti-CEA nanobody[5]	
VHH-C43	GAAGTTCAACTGGTTGAAAGCGGTGGTGGTCTGGTTCAGGCAGGCGGTAGTCTGACCCTGAGCTGTACCAGCAGCACCCTGACCTTTACACCGTATCGTATGGGTTGGTATCGTCAGACACCGGGTAAACAGCGTGATCTGGTTGCAGATATTAGCCCTGGTGATGGTAGCACCAAAAACTATGCAGGTTTTGCACAGG	Anti-CEA nanobody[6]	

	GTCGTTTTACCATTAGCCGTGATAATATCA AAAACACCGTT TACCTGCAGATGAACGATCTGAAACCGGA AGATACCGCAGTGTATTATTGTAATACCTA TGTTGCCTTTGTTGGTCGTGCACGTACCT GGGGTCAGGGCACCCAGGTTACAGTTAG CAGC	
VHH-CYT	CAGGTGCAGCTCGTGGAGACGGGGGGA GGCTTGGTGCAGCCTGGGGGGTCTCTGA GACTCTCCTGTGCAGCCTCTGGATTCACC TTCAGTAGCGTCTACATGAACTGGGTCCG CCAGGCTCCAGGGAAGGGGCCCGAGTG GGTCTCGCGTATTAGTCCGAATAGTGGTA ATATTGGGTATACAGACTCCGTGAAGGGC CGATTCACCATCTCCAGAGACAACGCCAA GAACACACTGTATCTGCAAATGAATAACC TGAAACCTGAGGACACGGCCCTGTATTAC TGTGCGATTGGTTTGAATTTGAGTAGTAG CTCCGTTAGGGGCCAGGGGACCCAGGTC ACCGTCTCCTCA	Anti-Spike protein nanobody[7]
proD-g10	CACAGCTAACACCACGTCGTCCCTATCTG CTGCCCTAGGTCTATGAGTGGTTGCTGGA TAACTTTACGGGCATGCATAAGGCTCGTA TAATATATTCAGGGAGACCACAACGGTTT CCCTCTACAAATAATTTTGTTTAACTTTGA ATTCAAAAGATCTTTAACTTTAAGAAGGAG ATATACAT	BBa_K1741014 and BBa_K1758100

a

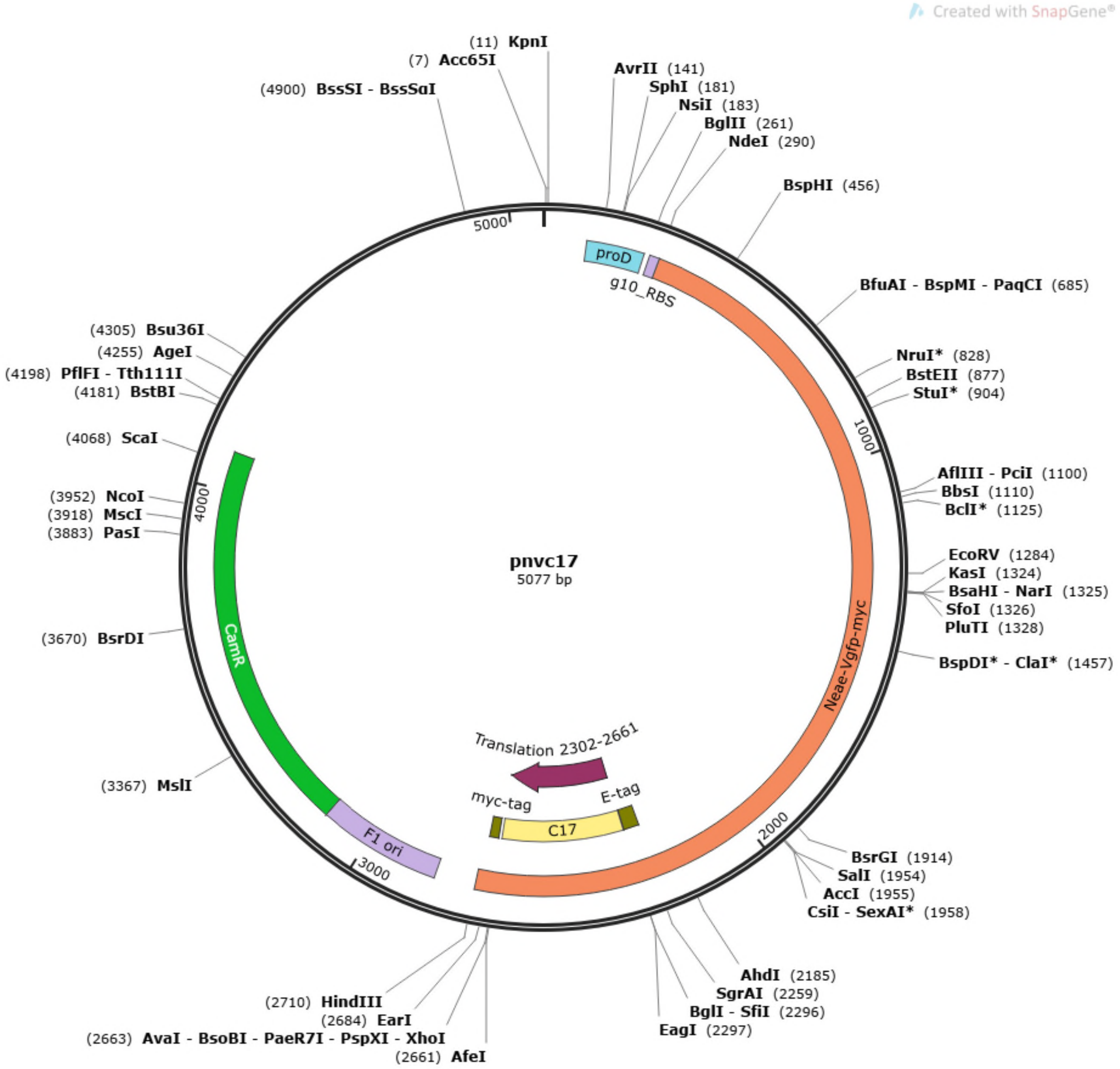
Created with SnapGene®
(11) KpnI
(7) Acc65I
(4900) BssSI - BssSαI
AvrII (141)
SphI (181)
NsiI (183)
BglII (261)
NdeI (290)
BspHI (456)
BfuAI - BspMI - PaqCI (685)
NruI* (828)
BstEII (877)
StuI* (904)
AflIII - PciI (1100)
BbsI (1110)
BclI* (1125)
EcoRV (1284)
KasI (1324)
BsaHI - NarI (1325)
SfoI (1326)
PluTI (1328)
BspDI* - ClaI* (1457)
BsrGI (1914)
SalI (1954)
AccI (1955)
CsiI - SexAI* (1958)
AhdI (2185)
SgrAI (2259)
BglI - SfiI (2296)
EagI (2297)
(2710) HindIII
(2684) EarI
(2663) AvaI - BsoBI - PaeR7I - PspXI - XhoI
(2661) AfeI
(3367) MslI
(3670) BsrDI
(3952) NcoI
(3918) MscI
(3883) PasI
(4068) ScaI
(4305) Bsu36I
(4255) AgeI
(4198) PflFI - Tth111I
(4181) BstBI
proD
910_RBS
Neae-Vgfp-myc
CamR
F1 ori
C17
myc-tag
E-tag
Translation 2302-2661
pnvc17
5077 bp
1000
2000
3000
4000
5000

b

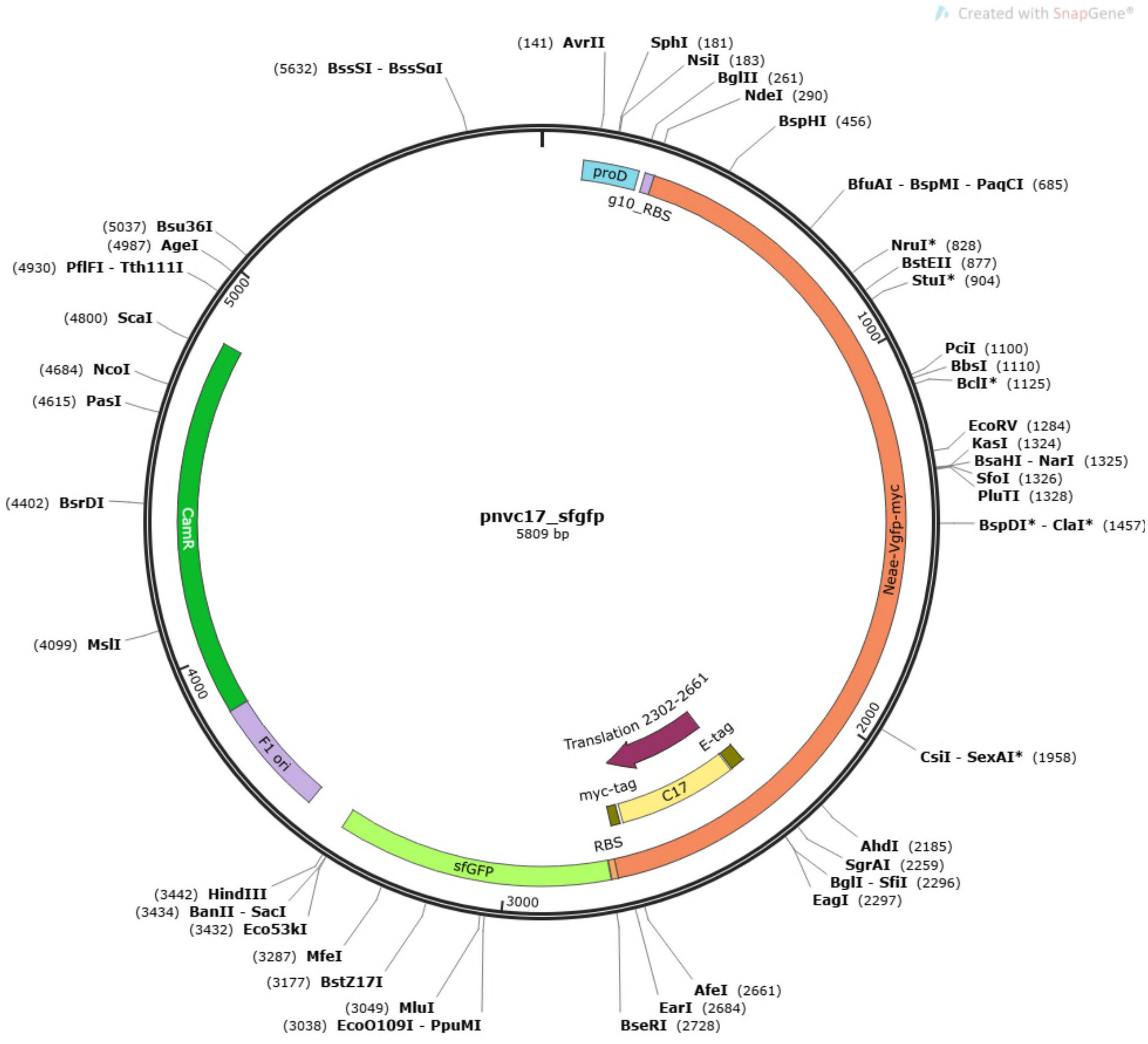

Figure S1. Plasmid map of (a) pNVC17 and (b) pNVC17_sfGFP plasmids. The promoter (proD), ribosome binding site (g10_RBS) and nanobody (C17) are interchangeable with other parts listed in Table S2.

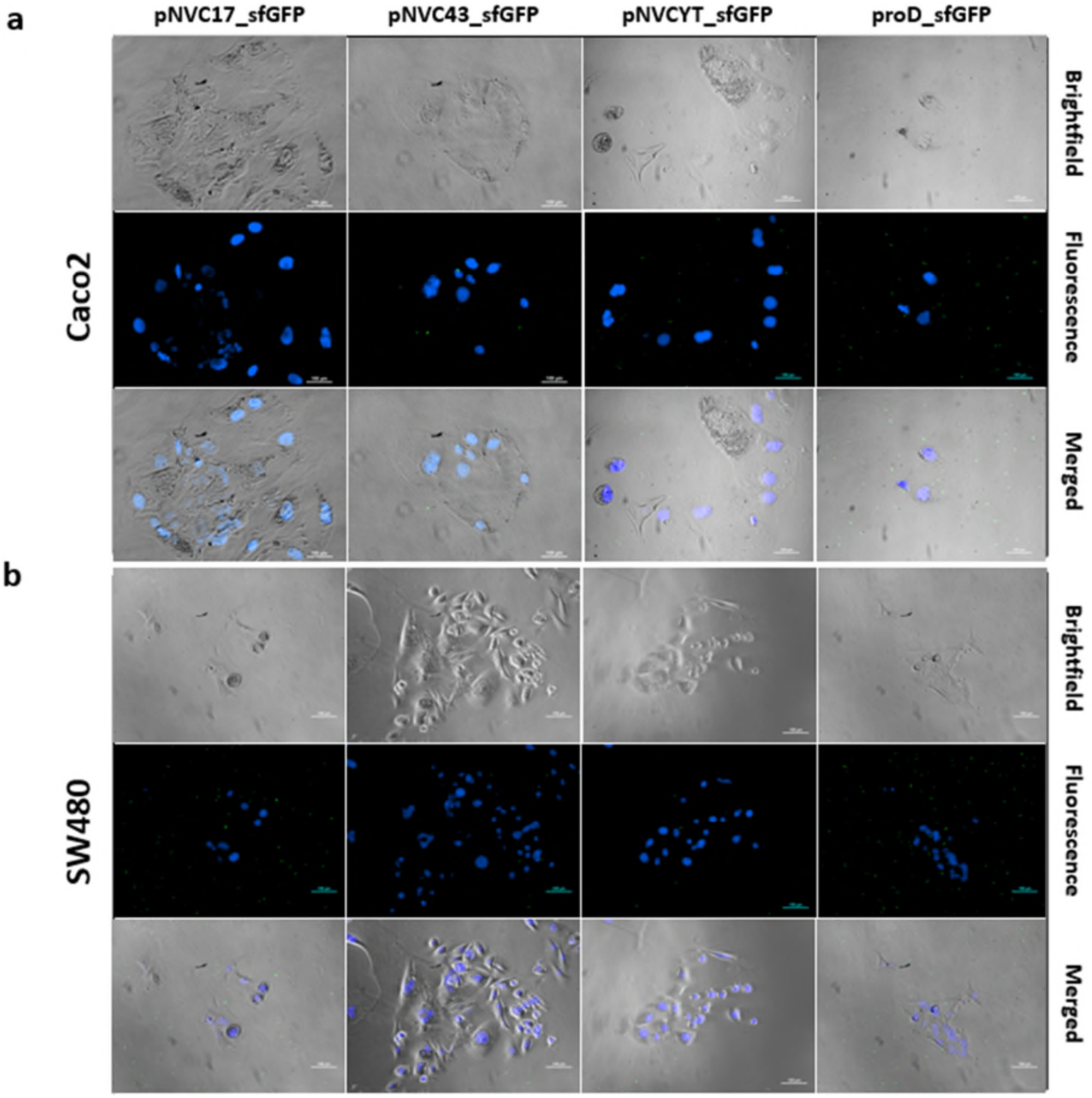

Figure S2. Time 0 hour (within ~5 minutes) incubation of engineered *E. coli* carrying different pNV plasmids with sfGFP (green) with two different colorectal cancer cell lines: **(a)** Caco2, a high CEA expressing cell line and **(b)** SW480, a low CEA expressing cell. Nuclei of cancer cells are stained with Hoechst dye (blue). Scale bar is 100 μm.

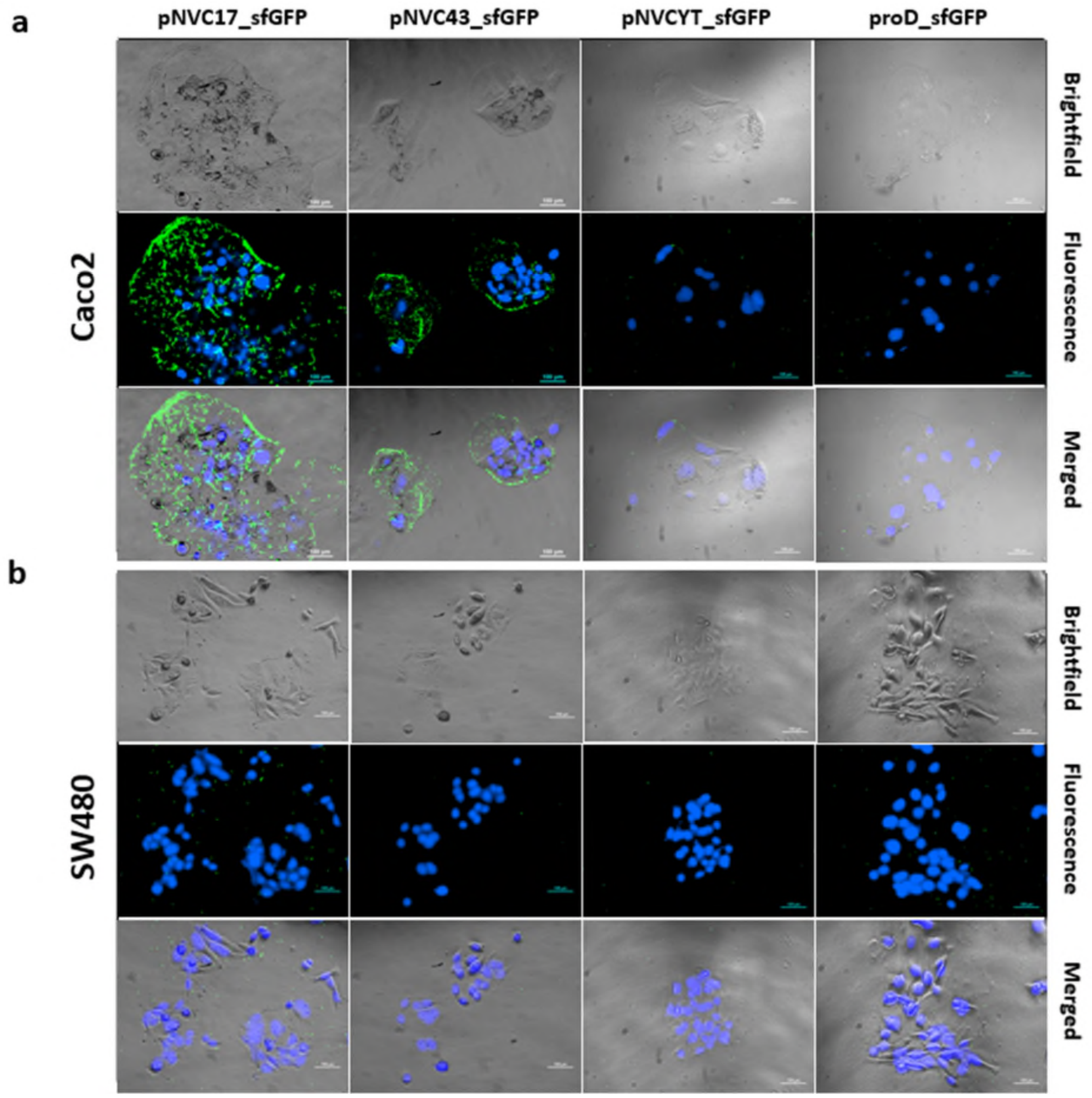

Figure S3. Time 4 hours incubation of engineered *E. coli* carrying different pNV plasmids with sfGFP (green) with two different colorectal cancer cell lines: **(a)** Caco2, a high CEA expressing cell line and **(b)** SW480, a low CEA expressing cell. Nuclei of cancer cells are stained with Hoechst dye (blue). Scale bar is 100 µm.

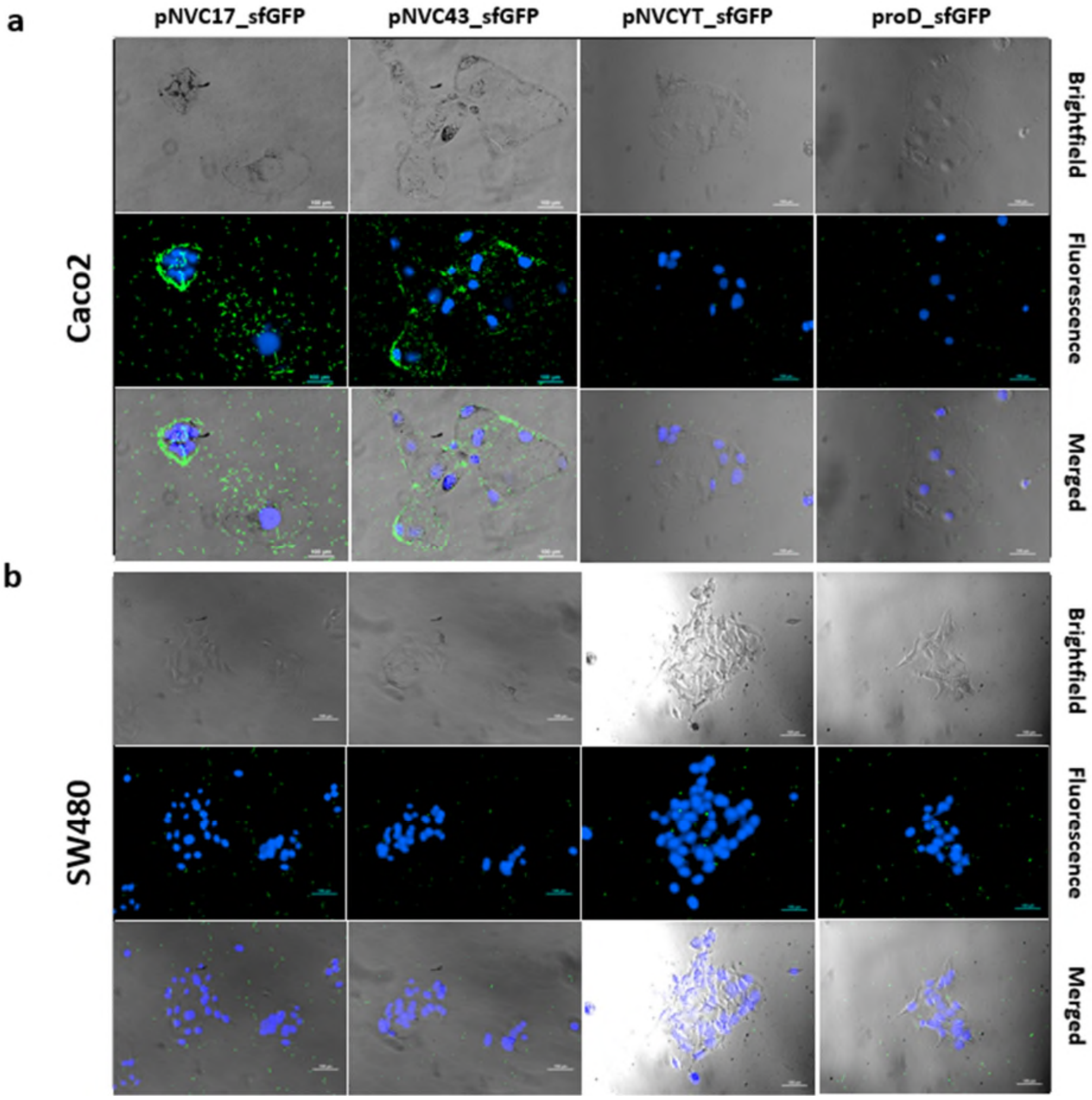

Figure S4. Time 8 hours incubation of engineered *E. coli* carrying different pNV plasmids with sfGFP (green) with two different colorectal cancer cell lines: **(a)** Caco2, a high CEA expressing cell line and **(b)** SW480, a low CEA expressing cell. Nuclei of cancer cells are stained with Hoechst dye (blue). Scale bar is 100 μm.

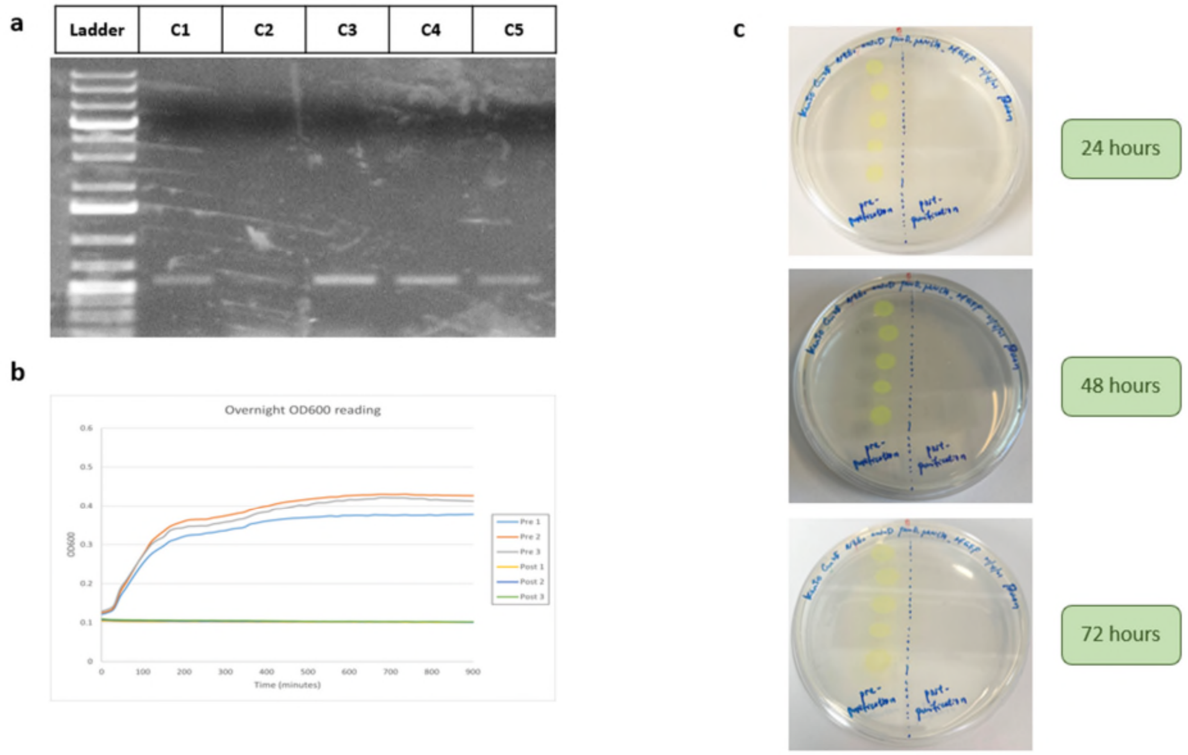

Figure S5. **(a)** Gel electrophoresis of PCR products from five different colonies (C1-C5) with successful *minD* knock out. The primers used were minD-HR-Kan-for and minD-HR-Kan-rev (Table S2), which anneal to the inserted Kanamycin cassette and the *minE* gene within the bacterial genome, respectively. The length of PCR product is 563 bp. The ladder used is a 1kb plus DNA ladder from NEB. **(b)** Overnight OD_{600} reading of pre-purification culture (pre 1, 2, 3) and post-purification culture (post 1, 2, 3) of minicells. Each line represents a single biological repeat. **(c)** Pre-purification and post-purification minicell culture plated on LB-Kan-Cm agar for 24 hours (top), 48 hours (mid) and 72 hours (bottom) at 37 °C.

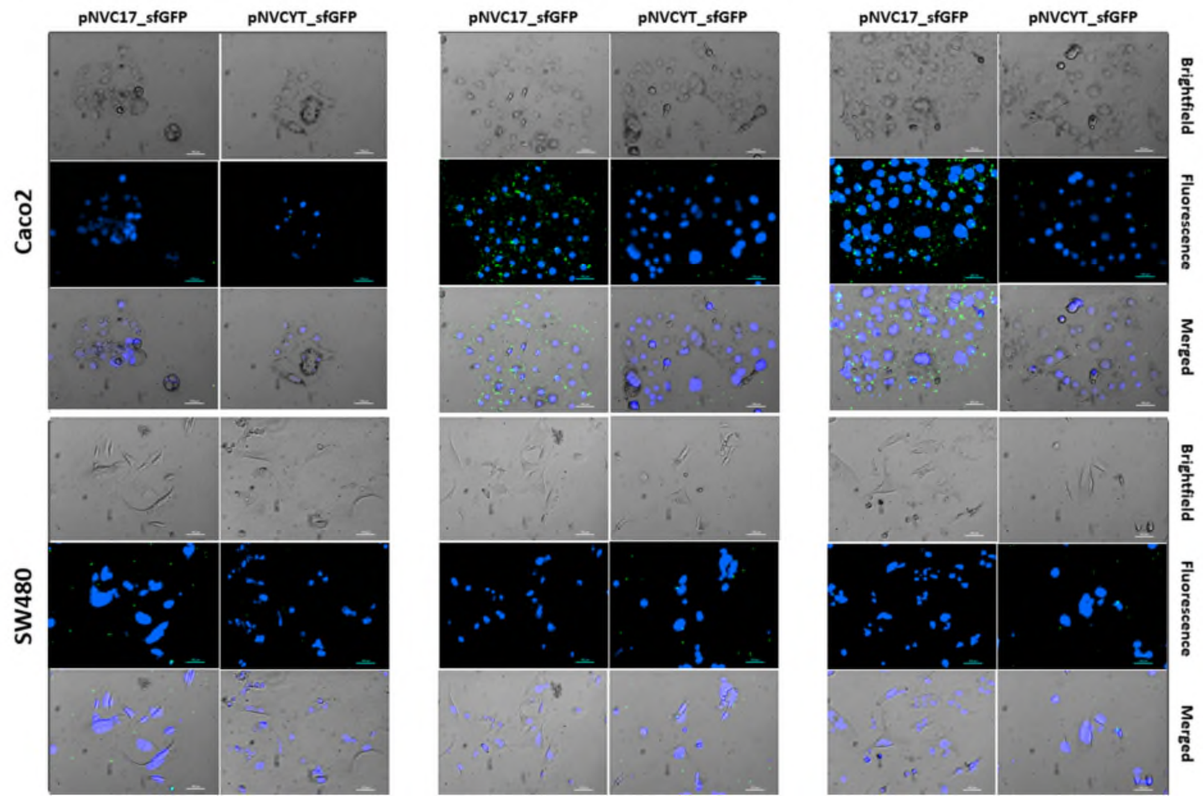

Figure S6. Time at 0 (left), 4 (mid) and 8 (right) hours incubation of engineered minicells carrying pNVC17_sfGFP or pNVCYT_sfGFP plasmids (green) with two different colorectal cancer cell lines: **(a)** Caco2, a high CEA expressing cell line and **(b)** SW480, a low CEA expressing cell. Nuclei of cancer cells are stained with Hoechst dye (blue). Scale bar is 100 μm.

.

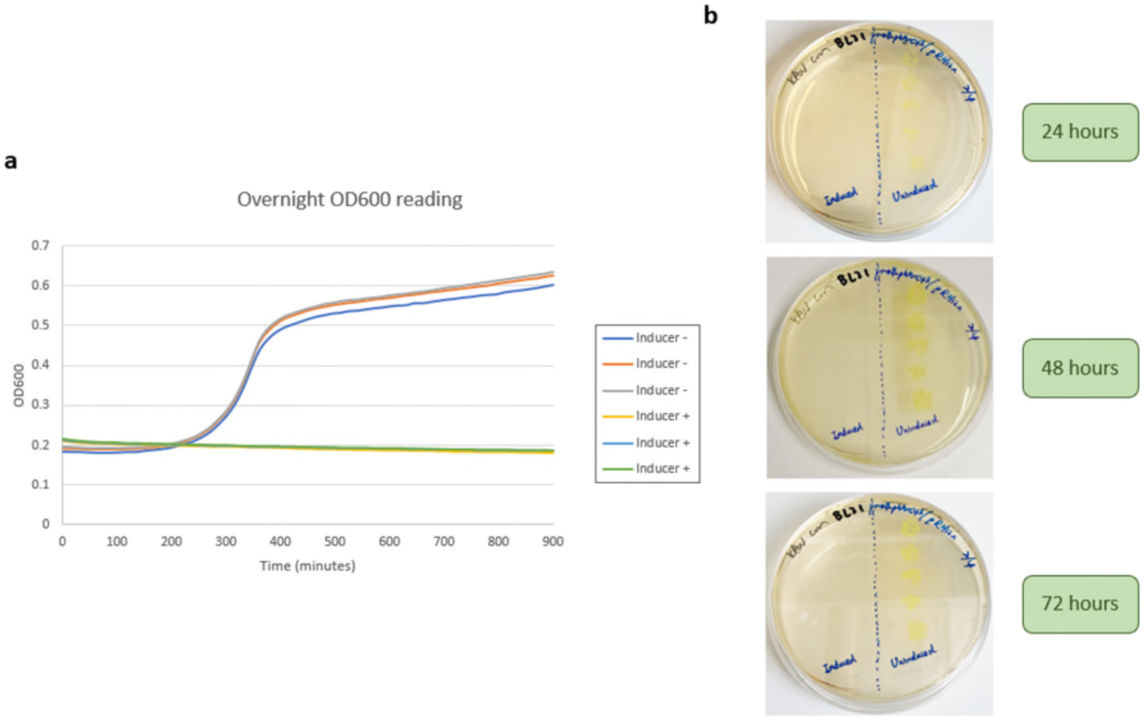

Figure S7. **(a)** Overnight OD600 reading of SimCell pYB1 carrying pNVC43_sfGFP after two hours induction of inducer cocktail X (inducer +) or without inducer (inducer -). Each line represents a single biological repeat. (b) Induced and uninduced SimCell pYB1 culture plated on LB-Kan-Cm agar for 24 hours (top), 48 hours (mid) and 72 hours (bottom) at 37 °C.

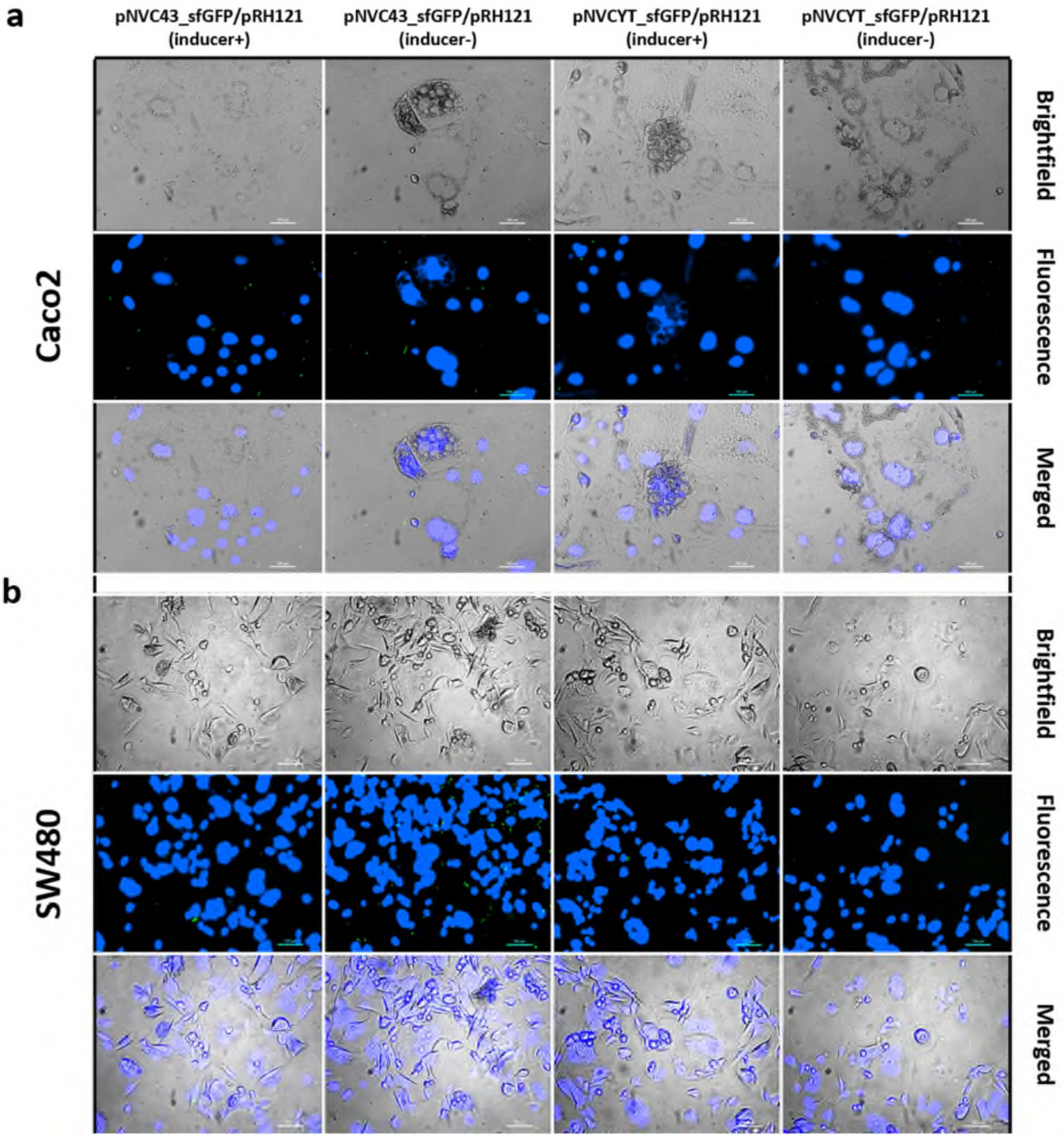

Figure S8. 0 hour (~within 5 minutes) incubation of programmed SimCell pYB1 carrying pNVC43_sfGFP or pNVCYT_sfGFP with (+) and without (-) the addition of inducer cocktail X (green). Bacterial cells under inducer+ are genome less and non-dividing SimCells. **(a)** Caco2 and **(b)** SW480 are the high CEA expressing and low CEA expressing colorectal cancer cell lines, respectively. Nuclei were stained with Hoechst dye (blue). Scale bar is 100 μm.

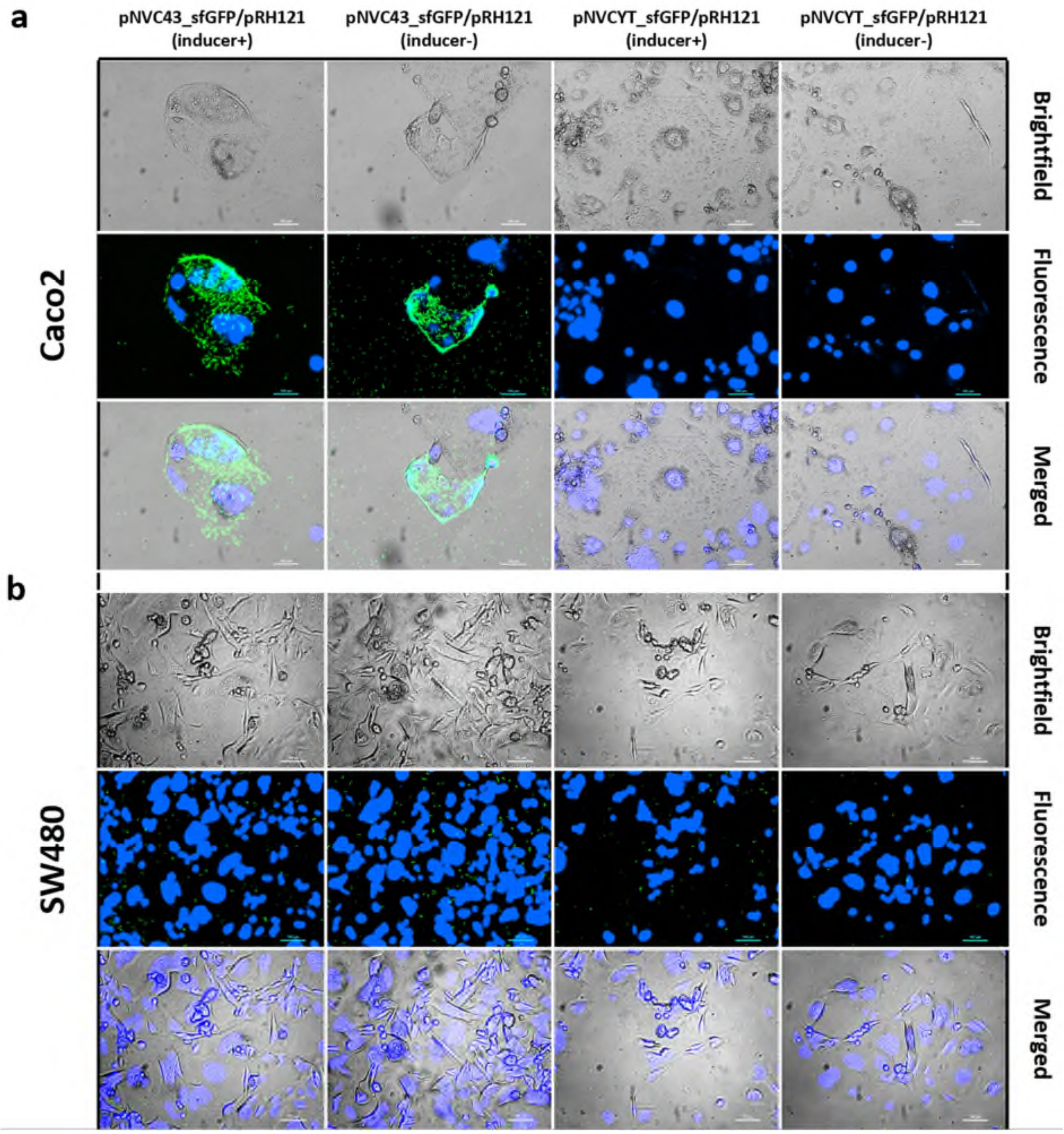

Figure S9. Time 4 hours incubation of programmed SimCell pYB1 carrying pNVC43_sfGFP or pNVCYT_sfGFP with (+) and without (-) the addition of inducer cocktail X (green). Bacterial cells under inducer+ are genome less and non-dividing SimCells. **(a)** Caco2 and **(b)** SW480 are the high CEA expressing and low CEA expressing colorectal cancer cell lines, respectively. Nuclei were stained with Hoechst dye (blue). Scale bar is 100 μm.

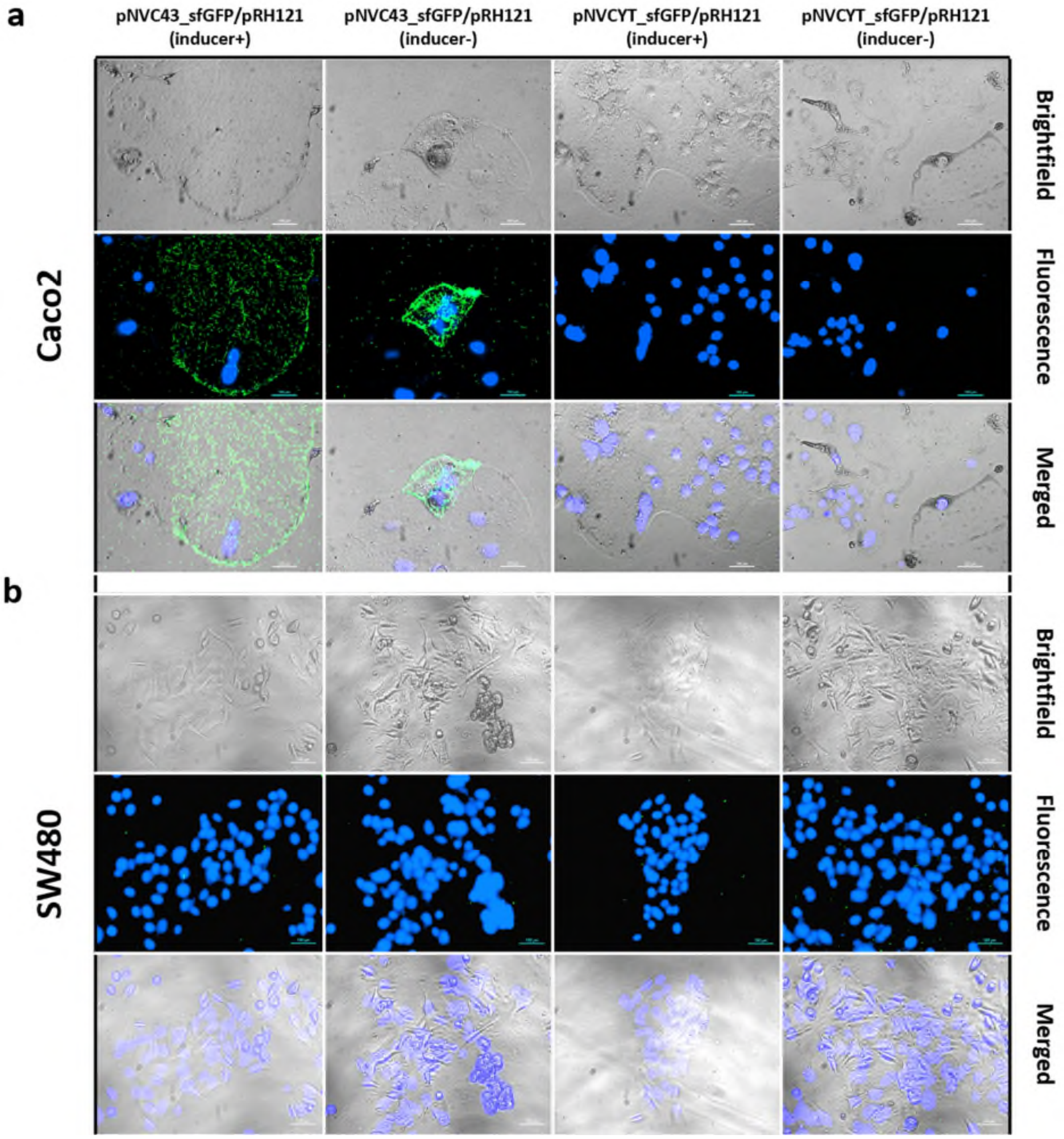

Figure S10. 8 hours incubation of programmed SimCell pYB1 carrying pNVC43_sfGFP or pNVCYT_sfGFP with (+) and without (-) the addition of inducer cocktail X (green). Bacterial cells under inducer+ are genome less and non-dividing SimCells. **(a)** Caco2 and **(b)** SW480 are the high CEA expressing and low CEA expressing colorectal cancer cell lines, respectively. Nuclei were stained with Hoechst dye (blue). Scale bar is 100 μm.

(Please access the video here:
https://1drv.ms/v/s!Atd6LwNkjPrwx158BYJ5FBiO6hxn?e=aladcC)

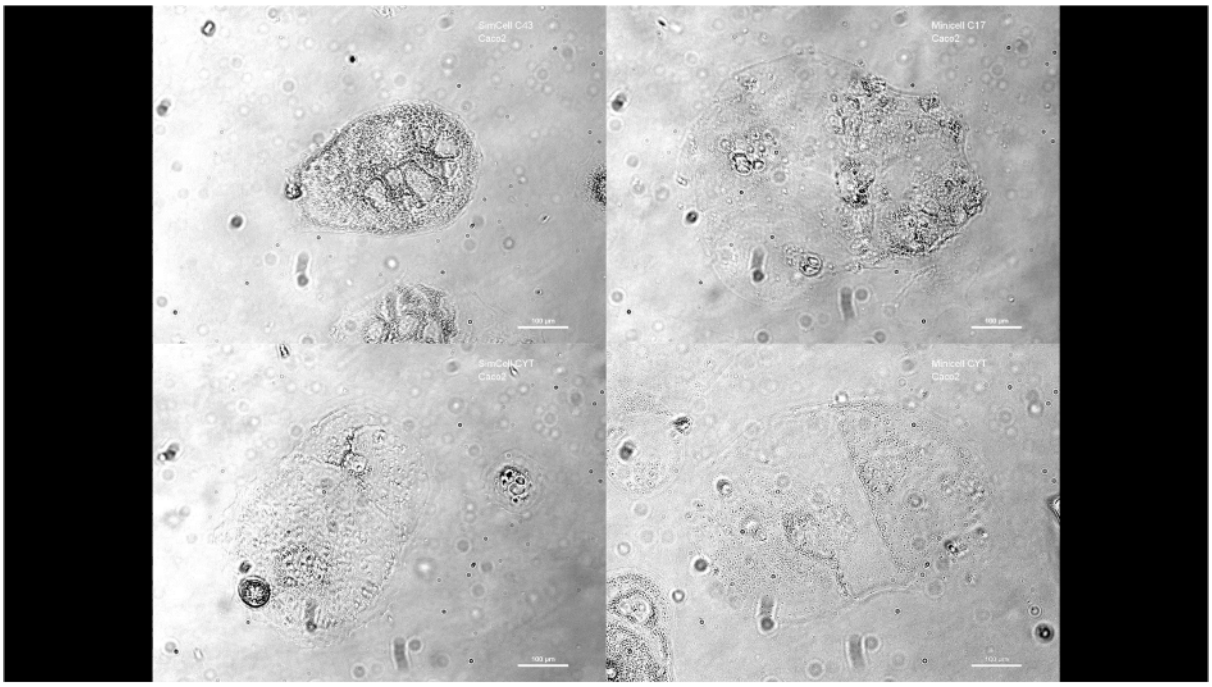

Movie 1. Time lapse video of Caco2 after 2 hours incubation with SimCell + pNVC43_sfGFP (top left); SimCell + pNVCYT_sfGFP (bottom left); minicell + pNVC17_sfGFP (top right); minicell + pNVCYT_sfGFP (bottom right). All cultures were washed thrice and added with fresh media supplemented with Ethidium homodimer prior to imaging. The duration of the experiment is labelled in hh:mm:ss format on the top left. SimCell and minicell are in green and the nuclei of Caco2 with compromised membrane were stained by Ethidium homodimer (red). Scale bar is 100 µm.

References

1. Pósfai, G. *et al.* Emergent Properties of Reduced-Genome Escherichia coli. *Science* **312**, 1044–1046 (2006).
2. Kylilis, N. *et al.* Whole-Cell Biosensor with Tunable Limit of Detection Enables Low-Cost Agglutination Assays for Medical Diagnostic Applications. *ACS Sens.* **4**, 370–378 (2019).
3. Fan, C. *et al.* Chromosome-free bacterial cells are safe and programmable platforms for synthetic biology. *Proc. Natl. Acad. Sci.* **117**, 6752–6761 (2020).
4. Jensen, S. I., Lennen, R. M., Herrgård, M. J. & Nielsen, A. T. Seven gene deletions in seven days: Fast generation of Escherichia coli strains tolerant to acetate and osmotic stress. *Sci. Rep.* **5**, 17874 (2015).
5. Kaliberov, S. A. *et al.* Adenoviral targeting using genetically incorporated camelid single variable domains. *Lab Invest* **94**, 893–905 (2014).
6. Behar, G. *et al.* Llama single-domain antibodies directed against nonconventional epitopes of tumor-associated carcinoembryonic antigen absent from nonspecific cross-reacting antigen. *FEBS J.* **276**, 3881–3893 (2009).
7. Hanke, L. *et al.* An alpaca nanobody neutralizes SARS-CoV-2 by blocking receptor interaction. *Nat. Commun.* **11**, 4420 (2020).
